# Nuclear Factor I genomic binding associates with chromatin boundaries

**DOI:** 10.1186/1471-2164-14-99

**Published:** 2013-02-12

**Authors:** Milos Pjanic, Christoph D Schmid, Armelle Gaussin, Giovanna Ambrosini, Jozef Adamcik, Petar Pjanic, Genta Plasari, Jan Kerschgens, Giovani Dietler, Philipp Bucher, Nicolas Mermod

**Affiliations:** 1 Institute of Biotechnology and Center for Biotecghnology UNIL-EPFL, University of Lausanne, Lausanne, 1015, Switzerland; 2 Ecole Polytechnique Fédérale de Lausanne and Swiss Institute of Bioinformatics, Lausanne, 1015, Switzerland; 3 Laboratory of Physics of Living Matter, Ecole Polytechnique Fédérale de Lausanne, Lausanne, 1015, Switzerland; 4 Peripheral Systems Laboratory, Ecole polytechnique Fédérale de Lausanne, Lausanne, 1015, Switzerland; 5 Present address: Swiss Tropical and Public Health Institute and University of Basel, Basel, Switzerland; 6 Present address: Selexis SA, Geneva, Switzerland; 7 Present address: ETH Zurich, Department of Health Science and Technology, Zurich, Switzerland; 8 Present address: Regenlab SA, Mont-sur-Lausanne, Switzerland

**Keywords:** Chromatin immunoprecipitation, Chromatin domain boundaries, Histone modifications

## Abstract

**Background:**

The Nuclear Factor I (NFI) family of DNA binding proteins (also called CCAAT box transcription factors or CTF) is involved in both DNA replication and gene expression regulation. Using chromatin immuno-precipitation and high throughput sequencing (ChIP-Seq), we performed a genome-wide mapping of NFI DNA binding sites in primary mouse embryonic fibroblasts.

**Results:**

We found that *in vivo* and *in vitro* NFI DNA binding specificities are indistinguishable, as *in vivo* ChIP-Seq NFI binding sites matched predictions based on previously established position weight matrix models of its in vitro binding specificity. Combining ChIP-Seq with mRNA profiling data, we found that NFI preferentially associates with highly expressed genes that it up-regulates, while binding sites were under-represented at expressed but unregulated genes. Genomic binding also correlated with markers of transcribed genes such as histone modifications H3K4me3 and H3K36me3, even outside of annotated transcribed loci, implying NFI in the control of the deposition of these modifications. Positional correlation between + and - strand ChIP-Seq tags revealed that, in contrast to other transcription factors, NFI associates with a nucleosomal length of cleavage-resistant DNA, suggesting an interaction with positioned nucleosomes. In addition, NFI binding prominently occurred at boundaries displaying discontinuities in histone modifications specific of expressed and silent chromatin, such as loci submitted to parental allele-specific imprinted expression.

**Conclusions:**

Our data thus suggest that NFI nucleosomal interaction may contribute to the partitioning of distinct chromatin domains and to epigenetic gene expression regulation.

NFI ChIP-Seq and input control DNA data were deposited at Gene Expression Omnibus (GEO) repository under accession number GSE15844. Gene expression microarray data for mouse embryonic fibroblasts are on GEO accession number GSE15871.

## Background

Nuclear factor I (NFI) was initially discovered as a cellular factor required for adenovirus DNA replication [1], where it binds to the origin of replication and recruits the viral DNA polymerase [2-4]. Subsequent studies showed NFI to be functionally and structurally indistinguishable from the sequence-specific CCAAT box-binding transcription factor CTF [5,6]. When bound to the promoter regions, NFI was found to act either as an activator or as a repressor of transcription [7-12]. NFI consists of a family of related transcription and replication factors that comprise the NFIA, NFIB, NFIC, NFIX polypeptides encoded by four paralogous genes in mammals [13,14], while orthologous NFI genes have been annotated in all examined vertebrate species [15,16]. In addition to the four NFI-coding genes, the diversity of this family of DNA binding proteins is further increased by the differential splicing of the gene transcripts [5,17]. However, all NFI isoforms share a homologous N-terminal domain responsible for the sequence specific DNA binding, while their C-terminal proline-rich regulatory domain differs between variants [18]. This C-terminal domain is required for either the activation or the repression of transcription, and in the case of the NFI-C isoforms, it has been shown to interact with nucleosomal histone H3, *in vitro* and on reporter promoters in transfected cells [19,20].

This ability of NFI-C to contact histone H3 has been proposed to alter the interaction of nucleosomal particles and DNA. For example, mouse mammary tumor virus (MMTV) LTR contains 6 nucleosomes that are being positioned after binding of NFI-C to its recognition site within the virus LTR [21,22]. This nucleosomal positioning is essential for the inducible response of the MMTV promoter to the glucocorticoid receptor. These findings indicated that NFI-C may directly regulate chromatin dynamics. Recent evidence also showed that NFI-C can act as a barrier protein that can stop the spreading of silent chromatin from yeast and human cell telomeres, and that it may thereby shield telomeric genes from heterochromatic silencing [20,23,24].

The different NFI isoforms are widely expressed, and knock-out of the individual genes yielded different phenotypes in mice, suggesting that its isoforms regulate distinct genes [14]. However, all NFI species bind similar dyad-symmetric TTGGC(N)_5_GCCAA sequence motifs as homo- or hetero-dimers [3,18,25,26]. As for other transcription factors, attempts to model the binding specificity from few in vitro-assayed binding sites was met with variable success [27,28]. However, an NFI weight matrix could be derived from a collection of over 10,000 binding sites selected using a SELEX-SAGE approach, and it was shown to provide reliable and quantitative estimates of NFI binding affinity and specificity in vitro [29]. However, to which extent this and other similar tools are capable of predicting in vivo interactions in the presence of chromatin and other DNA-binding proteins remains mostly untested.

In this work, we used chromatin immuno-precipitation coupled to next generation DNA sequencing (ChIP-Seq) to map NFI binding locations on the genome of murine fibroblasts. This indicated that the bioinformatics model of NFI sequence specificity accurately predicts binding occurrence in the cell, but that only a subset of the predicted binding sites are occupied. We also found that NFI preferentially associates with highly expressed genes, and that its binding is associated with active chromatin marks, even on binding sites occurring at distance from expressed genes, implying that it may directly control chromatin structure. Using a positional correlation of ChIP-Seq tags, we observed that NFI-containing complexes cover a nucleosomal length of DNA, unlike other DNA-binding proteins. Finally, we observed that NFI binding is often associated with the occurrence of genomic boundaries separating distinct chromatin structures. These findings suggest that the interaction of NFI with nucleosomal DNA may mediate chromatin domain barriers at natural genomic locations.

## Results and Discussion

### Genome-wide mapping of NFI binding sites

Using chromatin immuno-precipitation and a high-throughput DNA sequencing (ChIP-Seq) approach, we created a whole-genome map of NFI binding sites. This was performed using primary mouse embryo fibroblast (MEF) cells originated from wild-type (wt) and homozygous knock-out (ko) mice in which one of the NFI genes (*nf1-c*) was inactivated [30]. From wt cells, we obtained 14,358,325 reads, of which 9,771,440 (68.1%) could be mapped to unique positions in the genome. For ko cells, 10,809,703 out of 16,330,049 reads (66.3%) could be mapped to the genome. Our mapping efficiency is comparable to other ChIP-Seq experiments [31,32]. However, high multiplicity of tags mapping to the same genome position was observed, indicating that the material was PCR-amplified from a small founder population of DNA fragments. As a consequence, the total number of genome positions hit by one or more reads was reduced to 3,351,008 for wt cells, and 3,134,919 for ko cells.

A similar number of non-precipitated (input) genomic fragments were also sequenced as a control for potential biases in the distribution of genomic DNA fragments. From this DNA preparation, a lower fraction of tags (45%) could be uniquely mapped to the mouse genome, but there was also a lower degree of tag multiplicity. Overall, the 12,029,975 input sequences could be mapped to 4,104,241 unique positions.

*In vivo* NFI binding sites were identified with the ChIP-peak program of the ChIP-Seq tools, which is publicly available via a web interface (see Methods Section for details). This tool scans the genome in a sliding window of a fixed size and reports the center positions of genomic regions that are enriched in ChIP-Seq tags [33]. To get a comprehensive picture of NFI binding, we scanned the genome with different tag count thresholds. With thresholds of 5, 6, 7 and 8 tags, we obtained 14,487, 4,794, 1,642, and 701 peaks, respectively. These numbers document a low tag-coverage of binding sites, which may either reflect a low signal-to-noise ratio or the dilution of a true signal over a very large number of *in vivo* occupied binding sites. There are precedents of ChIP-Seq experiments with 5 to 10 tags per peak, *e.g.* Smad1 analysis in ES cells, where biologically meaningful sequence motif were generated from the peaks [34], justifying further analysis.

### Peak validation by motif enrichment test

**Figure 1:**
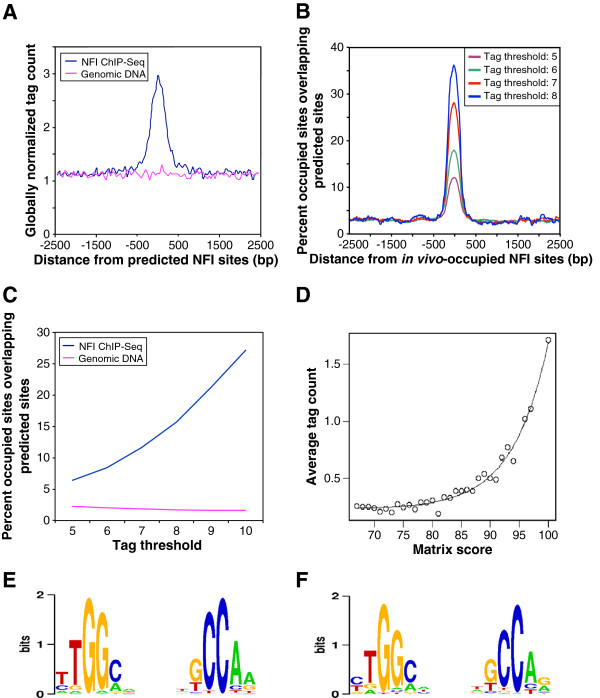
**Correlation of modeled NFI binding specificity to genomic binding site occupancy.** (**A**) Density of NFI ChIP-Seq tags in the vicinity of NFI sites predicted by the weight matrix derived from *in vitro* NFI binding studies. A score threshold of 90 yields 12,209 predicted sites on the mouse genome. Average ChIP-Seq tag counts were calculated in windows of 50 bp for a region of 2.5 kb on each side of the predicted sites. Tag counts were normalized globally as a fold-increase over the genome-wide average tag count in 50 bp windows. (**B**) *In vivo*-occupied NFI sites were defined with different tag thresholds: 8 tags yielding 701 sites, 7 tags – 1,642 sites, 6 tags – 4,794 sites, 5 tags – 14,487 sites. The percentage of in vivo NFI sites matching a predicted site was plotted as a function of the distance to the center of the *in vivo*-occupied sites in windows of 300 bp. NFI predicted sites were defined with the cut-off of 79. (**C**) Correlation of the tag threshold defining NFI site occupancy and weight matrix predicted sites, defined as fraction of in vivo sites that are overlapping a predicted site. (**D**) Exponential relation of the average tag count with the score of the position weight matrix. Tags were attributed to the predicted sites as follows: plus strand tags were attributed to the closest downstream predicted site if they were separated by less than 200 bp and minus strand tags were similarly attributed to the closest upstream predicted site. The average tag count was defined as the ratio of the overall tag number covering the predicted sites for a certain class and the number of predicted sites of the class. (**E**) In vitro NFI binding motif derived from 5579 NFI binding sequences of 25 bp in length obtained from SELEX-SAGE selection [29]. (**F**) In vivo NFI binding motif derived from 1265 NFI binding sequences of 200 bp in length, obtained from the ChIP-Seq experiment.

In view of the low tag coverage of extracted peaks, we first wished to obtain evidence that the peaks were biologically meaningful. To this end, we analyzed the distribution of the NFI binding site motifs around the peak center positions in the various peak lists using the SELEX-based weight matrix shown in Additional file 1: Figure S1. The results were ensuring, as we observed a narrow peak centered at the estimated binding site position at all thresholds (see Figure 1A and 1B and Additional file 1: Figure S2 and S3). With the highest tag threshold applied (8 tags), nearly 40% of the resulting peaks contain an NFI binding motif near the center position, which is comparable to what was commonly observed in other ChIP-Seq experiments. We thus concluded that most of these peaks are real binding sites. An almost four-fold lower motif enrichment of about 10% was observed with a tag threshold of 5 (see Figure 1B), suggesting that the corresponding peak collections may be contaminated with a larger proportion false positives. Nevertheless, in absolute numbers this peak collection is estimated to contain about 5 times more true sites than the peak list obtained with a threshold of 8 tags. In order to choose an appropriate peak threshold, we followed the guidelines of the ENCODE consortium [35], which require that a known binding motif of a transcription factor should be at least four-fold enriched and occur in at least 10% of the peak regions extracted from corresponding ChIP-Seq data. With our data, the lowest tag threshold meeting these criteria was 6 (enrichment factor 5.3 and motif occurrence frequency 17.8%, see Figure 1B). We thus used this peak threshold for all further analyses unless specified otherwise.

The peak finding method applied here does not use input control data for background subtraction nor does it rank peaks by P-values based on a statistical model. As this might affect the quality of the resulting peak sets, we repeated the peak finding step with the widely used MACS program [35]. The resulting peak lists were then evaluated with a particular motif enrichment plot used in a recent benchmarking paper for the same purpose [36]. ChIP-peak was found to outperform MACS with a rather large margin, especially if the latter program was used with default parameters (see Additional file 1: Figure S4). Regardless of the threshold, the peak lists obtained with ChIP-peak always contained more NFI motifs than those obtained with MACS. Somewhat better results were obtained when a tag shift of 150 bp was given as an input parameter to MACS (by default, MACS estimate this parameter from the data). Even better performance was observed when the input control was omitted. Usage of an input control thus did not improve performance of the peak finder. Based in this observation, we considered it unlikely that another peak finding method would perform substantially better on our data than ChIP-peak with optimized parameter settings.

### In vivo NFI binding sites partially overlap predicted binding sites

We next assessed whether NFI *in vivo* binding sites specificity correlates with binding site predictions based on a weight matrix model determined from *in vitro* binding assays using various analytical strategies. To this end, we first scanned the mouse genome with the NFI weight matrix previously established from high-throughput SELEX-SAGE assays (see Additional file 1: Figure S1A). This matrix was shown previously to accurately compute relative *in vitro* binding affinities [29]. The genome scan resulted 12,209 predicted high-affinity binding sites with a matrix score ≥90 (arbitrary score units, see Additional file 1: Figure S1B). Almost two million sites were found with a score threshold of 67, the lowest value for which *in vitro* binding activity was observed. The average counts of ChIP-Seq tags showed a substantial enrichment surrounding the center of the predicted NFI sites while no increased tag frequency was observed from the non-precipitated genomic input DNA (Figure 1A). When using lower matrix score cut-offs (85 or 80, yielding 61,492 and 231,146 predicted sites, respectively), a gradual reduction of the correlation was noted between the predicted sites and ChIP-Seq tags (see Additional file 1: Figure S5). We conclude that low scoring predicted sites are less frequently occupied by the protein *in vivo*, as would be expected from their lower predicted affinity.

Next, we focused on the 708 most highly occupied *in vivo* NFI sites, which correspond to a threshold of at least 8 tags, as nearly 40% of these sites colocalized with a medium-to-high scoring predicted binding sequence (weight matrix score ≥79; Figure 1B). We next wondered whether the remaining 60% co-localize with lower affinity binding sites. Lowering the score threshold to 67 increased the peak maximum to about 60%, but it simultaneously raised the background frequency to nearly 20% (see Additional file 1: Figure S6). Consequently, there was no net gain in motif enrichment when the weight matrix cut-off value was lowered. Thus, about half the highly occupied *in vivo* NFI sites do not co-localize with a recognizable NFI binding motif.

### The chromatin context does not change NFI intrinsic binding specificity

The partial overlap between binding sites predicted from an *in vitro* specificity model and *in vivo* binding sites determined by ChIP-Seq could be explained in at least two different ways. For instance, the *in vivo* binding specificity may be similar but not identical to that for naked DNA. For instance, interactions with histones and other chromatin proteins could alter the DNA structure in a way that would change the relative affinity of the NFI protein to different target sequences. Alternatively, the intrinsic sequence specificity may not be influenced by the chromatin context, but other mechanism may interfere with the NFI recruiting process, such as competition with other transcription factors, or the indirect recruitment of NFI by interactions with other DNA binding proteins.

To discriminate between these two hypotheses, we first we analyzed the correlation between weight matrix scores and ChIP-Seq tag coverage at the highest possible resolution. To this end, the tag coverage of all predicted NFI sites with scores ≥67 was determined as follows. Each plus strand tag was attributed to the closest downstream predicted site if such a site occurred within 200 bp. The minus strand tags were attributed to the closest upstream predicted site applying the same distance constraint. We then grouped the predicted sites by matrix score, and computed the average tag coverage for each score class. The tag count was found to increase exponentially with the weight matrix score (Figure 1D), which is consistent with the fact that the weight matrix scores were defined to have a log-linear relationship to the actual binding affinity [28,29]. The almost perfect correlation between predicted score and average tag coverage speaks against chromatin-induced changes of the *in vitro* determined binding specificity.

We then tried to directly compare the *in vivo* and *in vitro* binding specificities by generating a new weight matrix from the ChIP-Seq data with the same computational method that was used to generate the *in vitro* binding specificity matrix (shown as a sequence logo in Figure 1E). This logo was derived from 5,579 25bp-long NFI binding sequences obtained with a SELEX-SAGE experiment [29], using the hidden Markov model-training program MAMOT (Schütz and Delorenzi 2008) to iteratively optimize a weight matrix model starting from the consensus sequence TTGGCNNNNNGCCAA. Applying the same procedure to 4,794 200bp-long sequences centered on the ChIP-seq peaks obtained with a tag threshold of 6, we obtained the logo shown in Figure 1F. The sequence logos corresponding to the *in vitro* and *in vivo* binding specificity are almost identical, with the exception of the first and last nucleotide positions (Figure 1E and 1F and Additional file 1: Figure S6B).

In parallel, we carried out motif discovery with the program peak-motifs from the RSA-tools [36], which yielded an NF1-like motif from all peak lists. In addition, an AP1-like motif was also obtained from the peak lists obtained with thresholds of 6, 7 and 8 (see Additional file 2: Table S1). Taken together these findings suggest that other factors than chromatin accessibility may account for the imperfect overlap between predicted and observed *in vivo* binding sites. In particular, AP1 complexes may be involved in the indirect recruitment of NFI protein to those target sites which lack a canonical NFI motif.

### Genomic distribution of NFI sites relative to genes

Of the 4,794 binding sites identified with a tag threshold of 428 occurred within 5 kb upstream of genes, 106 in protein coding exons, and 232 within 5 kb downstream of genes (see Additional file 2: Table S2). As expected, the majority of binding sites occurred in introns and in intergenic regions (1,966 and 2,138). However, these NFI-bound sites were not randomly distributed across the genome, as they appeared more frequently around genes. Upstream regions, 5’UTR exons and 3’UTR exons contained the highest binding sites densities, with about 3.9, 12.3 and 3.3 binding sites per Mb, respectively, while intergenic regions had the lowest density (about 1.3 binding sites per Mb; see Additional file 2: Table S2). Overall, NFI genomic distribution resembles that of many other DNA binding proteins assayed by ChIP-Seq [34], including the insulator protein CTCF, to which we will refer below in another context. The distribution of NFI sites is however markedly different from that of classical promoter-associated transcription factors such as c-Myc.

### Genome-wide mapping of NFI binding sites in NFI-C ko cells

We carried out similar NFI-C ChIP-Seq experiment with NFI-C ko MEF cells. Analysis of extracts of such cells showed a complete absence of the 55 KDa polypeptide corresponding to the major splice variant of NFI-C, and a significantly reduced level of other NFI species when analyzed by Western blotting (see Additional file 1: Figure S7A and S7B). ChIP-Seq tag counts from NFI-C ko cells showed slightly reduced occupation of NFI predicted sites in comparison to the wild type tag set (see Additional file 1: Figure S7C), which was confirmed by quantitative PCR for several randomly chosen high-scoring predicted sites (data not shown). Sequence motif analysis of the strongest *in vivo* sites from wt and ko MEF using MEME [37] revealed a similar most prominent sequence logo (see Additional file 1: Figure S6), indicating similar binding specificities for all NFI species, as expected from previous reports. By extracting peaks and comparing the peak lists from the two data sets, we found little differences between *in vivo* occupancy patterns. We conclude that the same sites are occupied by an NFI protein family in NFIC wt and ko cells, but obviously not always by the same NFI species.

### Determination of the length of NFI binding complexes using ChIP-Seq

High throughput sequencing of DNA fragments may occur from both extremities of the DNA fragments, and the corresponding sequence tags will be mapped to opposite (plus or minus) DNA strands. This was used to analyze the length of the DNA that is occupied by the precipitated protein complex, assuming that DNA cleavage occurs preferentially outside of cross-linked protein-DNA complexes. Thus, genome-wide positional correlations between the plus and minus strand-mapping tags should reveal the average size of the DNA being protected by the protein complex when single-end sequencing is used, as performed in this study (see Additional file 1: Figure S8).

**Figure 2:**
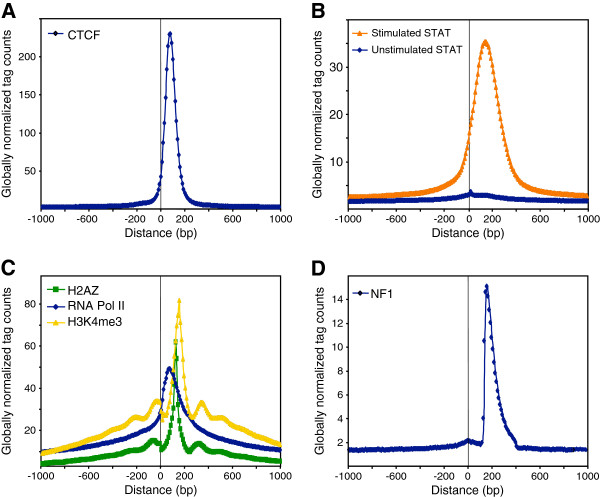
**Positional correlation of tags mapping on plus and minus strand from other ChIP-Seq experiments.** Co-localized tag counts greater than 10 were set to 10 and tags overlapping repeated DNA sequences, as defined by RepeatMasker, were filtered out. Tags mapping on the plus strand were centered to position 0 and the distribution of tags mapping on the minus strand is displayed as a function of the distance to the centered plus strand tags, using ChIP-Seq datasets generated for CTCF (panel **A**), STAT1 from stimulated and unstimulated cells (**B**), the H2AZ histone variant, RNA Pol II, and histone H3 tri-methylated on lysine 4 (H3K4me3) (**C**), or NFI (**D**).

We separated NFI ChIP-Seq tags into datasets comprising either the plus or minus strand-mapping tags, yielding similar tag counts (4,890,670 and 4,880,749, respectively), indicating that Illumina sequencing is not biased in this respect. Genome-wide positional correlation between the plus and minus strand tags was made by assessing the frequency of minus tags at varying distances from the plus tags. The position-specific minus tag frequencies were divided by the genome-wide average frequency for normalization purposes. As a basis for comparison, we also generated such profiles for the CTCF and STAT-1 transcription factors, using previously reported ChIP-Seq datasets [31,38]. The resulting distributions appear to be Gaussian, with the peak maximum at 80bp for CTCF and 140bp for STAT1 (Figure 2A,B). The larger spacing between plus and minus strand tags seen with STAT1 is consistent with the previous observation that STAT-1 can form dimers or trimers that occupy larger portions of the DNA [39,40]. We also assessed the STAT1 control ChIP-Seq dataset generated from cells not stimulated with interferon, where STAT1 is not activated and does not enter into the nucleus. This dataset yielded a uniform distribution (Figure 2B), as expected from the absence of STAT-DNA interactions. The distributions of the histone H3 trimethylated on lysine 4 (H3K4me3) and of the H2AZ histone variant showed multiple peaks corresponding to mono-, di- and tri- nucleosomes, with a main peak corresponding to a mononucleosomal length of DNA of about 150-200bp (Figure 2C). A similarly analyzed RNA Polymerase II profile showed a more diffuse and tailing distribution, which might reflect the progression of the polymerase along transcribed genes (Figure 2C).

In sharp contrast, NFI data set analysis revealed an asymmetric peak (Figure 2D), with a steep increase at 150bp and a rather shallow decrease ranging between 150bp to 250bp. This type of correlation was thus substantially different from what was observed for the other transcription factors. One of the possible explanations of the asymmetrical plus and minus tag peak mediated by NFI might result from NFI-histone interactions. A specific feature of NFI proteins is their ability to interact with nucleosomal histone H3 and to affect the nearby chromatin structure [19-22]. Therefore, the cross-linked genomic DNA might be cleaved preferentially between nucleosomes, yielding mostly mono-nucleosomal DNA, and a complex formed by the interaction of NFI with a nucleosome would withstand genomic DNA fragmentation. An alternative explanation might be that this distribution reflects the actual fragment length distribution after all selection steps: sonication, size selection by electrophoresis, and a possible intrinsic size-selection of the sequencing technology.

This was assessed by performing the positional correlation of the plus and minus tags from the sequencing of fragmented but non-immunoprecipitated genomic DNA, which yielded a more diffuse profile with a maximum at 180 bp, suggesting that chromatin sonication resulted mostly in mononucleosomal particles (see Additional file 1: Figure S9). This interpretation was confirmed by releasing the genomic DNA from cross-linked chromatin proteins after sonication, and by directly measuring the length of DNA fragments using scanning atomic force microscopy (AFM). This assay revealed an overrepresentation of DNA fragments comprised between 50 and 250 bp, with most sequences ranging between 100 and 160 bp (see Additional file 1: Figure S10), implying that the DNA population used for immunoprecipitation indeed consisted mostly of mononucleosomal complexes.

Mapping of the polarity of tags surrounding NFI predicted sites revealed symmetrically distributed profiles of the plus and minus strand tags around the centers of the binding sequence dyad (see Additional file 1: Figure S5). Well-positioned tag distributions were most prominent for the high-scoring sites, which often correspond to frequently occupied binding sites, implying that this pattern directly results from binding site occupancy. The distribution maxima were clearly separated by approximately 150 bp. These observations are consistent with the interpretation that NFI proteins may co-precipitate with nucleosomal complexes occupying around 150 bp of DNA, although other interpretation remain possible. Our proposed model of NFI interaction with the DNA in a nucleosomal protein complex is shown in the Additional file 1: Figure S11.

### NFI associates with open chromatin marks and chromatin domain boundaries

**Figure 3:**
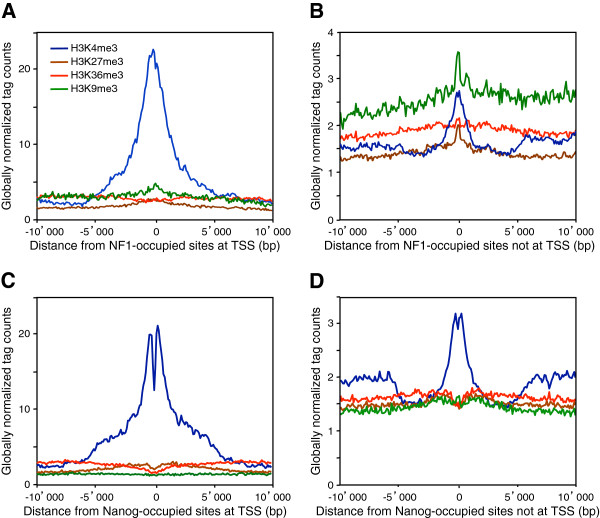
**NFI associates with histone modification H3K4me3.** In vivo NFI sites were defined with the threshold of 6 tags yielding 4,794 sites. In vivo NFI sites were separated into two categories: one group (808 sites) mapping to regions +/− 5 kb from transcription start sites (TSS) and another group outside these regions (3,986 sites). The average ChIP-Seq tag counts for different histone modifications were calculated in windows of 100 bp for a region of 10 kb up- and down- stream of the in vivo NFI sites. Tag counts were normalized globally, as a fold-increase over the genome average tag count in a window of 100 bp. In vivo Nanog sites were separated into two categories: mapping into regions +/− 5 kb from transcription start sites (TSS) and outside these regions. The average ChIP-Seq tag counts for different histone modifications were calculated as for NFI. (**A**) In vivo NFI sites inside TSS +/− 10 kb. (**B**) In vivo NFI sites outside TSS +/−10 kb. (**C**) In vivo Nanog sites inside TSS +/− 10 kb. (**D**) In vivo Nanog sites outside TSS +/− 10 kb.

To test whether NFI may preferentially associate with the markers of specific chromatin structure, we used available ChIP-Seq data of histone modifications in MEF cells [41]. We first separated *in vivo*-occupied NFI sites occurring at promoters, within 5kb upstream or downstream from the RefSeq annotated transcriptional start sites (TSS), from those mapping elsewhere. Out of 14,487 occupied sites, 2,040 map within 5 kb of RNA polymerase II TSS in both directions. As expected, these TSS-associated sites were highly correlated with the H3K4me3 modification known to occur at the promoters and enhancers of expressed genes (Figure 3A) [41-43]. Surprisingly, *in vivo* NFI sites outside TSS were also associated with this modification, but to a smaller extent (Figure 3B). The association with H3K4me3 suggested that NFI binding might be associated to the deposition of this histone mark, for instance at distal transcriptional enhancers [38] or at chromatin barriers [44]. Alternatively, it may occur at un-annotated TSS corresponding to e.g. unknown transcripts and/or at the TSS of miRNAs, as 3 NFI sites were found to overlap the promoters of primary miRNA transcripts in the mouse genome (see Additional files 1: Figure S12 and 2: Table S3) [45]. The levels of H3K36me3 marker of transcribed regions increased with increasing distance from the TSS-proximal *in vivo* NFI sites (Figure 3A), which is indicative of the active transcription of these genes [41]. The level of H3K36me3 and H3K4me3 were also slightly elevated in the vicinity of NFI binding sites mapping outside TSS, but they displayed a pattern that was distinct from the ones observed at protein-coding or miRNA genes (Figure 3B and Additional file 1: Figure S12), suggesting that they do not correspond to non-annotated genes. For comparison, we provide similar graphics for another transcript factor (Nanog) and corresponding histone marks in ES cells (Figure 3C-D). The overall picture is similar with one notable exception: The major H3K4me3 peak is bimodal, with a minimum right at the center of the binding region. The different peak shapes lend further support to our hypothesis that NFI binds to nucleosomes, in contrast to most other transcription factors, including Nanog, which bind to nucleosome-free regions.

**Figure 4:**
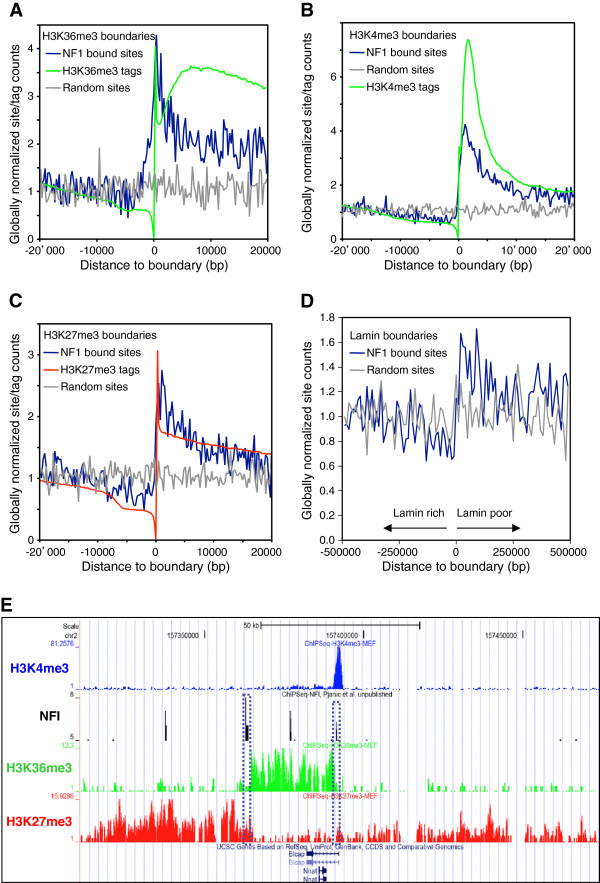
**NFI binding is associated with chromatin and nuclear lamina boundaries.** The mouse genome was partitioned into histone modification-poor and enriched segments, and the genomic positions of boundaries featuring transitions from modification-poor to enriched regions were centered and aligned at position 0 of the X-axis, from (**A**) 14,632 H3K36me3 boundaries, (**B**) 31,662 H3K4me3 boundaries (**C**) 29,122 H3K27me3 modification boundaries. The distribution of 4,794 in vivo NFI sites was compared with the distribution of 4,794 randomly chosen genomic positions sampled from each chromosome according to the number of occupied NFI sites. The average numbers of NFI or random sites were normalized globally with the genome-wide average number of sites in 250 bp window. The distribution of NFI and random sites was also aligned to the anchor spots of DNA to the nuclear lamina using 2 kb windows (**D**), where the arrows indicate the poor and enriched genomic regions for the nuclear lamina lamin B component. (**E**) Example of NFI in vivo binding sites located at the boundaries of open and close chromatin markers, H3K36me3 and H3K27me3, in mouse embryonic fibroblasts. NFI binding sites define the region of open chromatin surrounding the Blcap and Nnat gene locus. NFI sites at the boundary positions are enclosed with a dotted line.

It has been shown that NFI may act to prevent the propagation of silent chromatin structures and thereby to promote the formation of chromatin domain boundaries at synthetic yeast and human cell telomeres [20,23,24]. We tested whether this may also occur at natural genomic locations by partitioning the mouse genome into genomic regions enriched or depleted of specific chromatin modifications. Chromatin domain boundary (or barrier) positions featuring sharp transitions of histone modifications were sorted and compared to the localization of 14,487 NFI-occupied sites. As a control set, we randomly selected 14,487 genomic positions in the mouse genome, taking from each chromosome the same number of random loci as of the NFI-occupied sites. NFI-bound sites were found to be significantly enriched near the boundary regions of three different histone modifications characteristic of expressed, accessible or silent chromatin, respectively, namely H3K36me3, H3K4me3 and H3K27me3, while randomly selected control genomic sequences were distributed uniformly around these boundary positions (Figure 4A-C).

From the locations of the distribution maxima, NFI sites appeared to be located on the histone methylation-enriched side of the boundaries. To address further this possibility, we calculated the distances from the boundary to the nearest NFI or random control site in both orientations relative to the boundary. The distribution plot of the first 2,000 smallest distances indicated that NFI often bound the modification-rich side for all three types of histone H3 modifications when compared to the randomly selected sites (see Additional file 1: Figure S13). An NFI site was found within 2.5 kb of the modification-enriched side of 823 (2.8%), 549 (3.8%) and 1391 (4.4%) of the H3K27me3, H3K36me3 and H3K4me3 boundaries, respectively. An example of NFI binding a boundary separating an open chromatin domain from neighboring closed chromatin consists of the Blcap and Nnat imprinted genes locus, where a H3K27me3-poor and H3K36me3-enriched domain is bracketed by two NFI-bound sites (Figure 4E). Additional examples of NFI-associated boundaries are shown in the Additional file 1: Figure S14.

The association of NFI with boundaries suggested that it may indeed contribute to the formation of chromatin barriers, but that its binding may not be sufficient to promote boundaries at all genomic loci, as occupied binding sites also occur within chromatin domains (Figure 4E and Additional file 1: Figure S13). NFI-bound chromatin domain boundaries were often but not always associated with promoters. For instance, one of the NFI-bound boundaries was found to coincide with the active Blcap gene promoter, as also indicated by a high incidence of H3K4me3 modification (Figure 4E). However, the other domain boundary located 20 kb downstream of the Blcat gene showed a relatively stronger NFI-binding site, which corresponds to a consistently stronger histone H3K27me3 and H3K36me3 modification boundary that does not bear the H3K4me3 marks of promoters. Similar observations were made at other genomic loci, suggesting that NFI may separate transcribed and/or accessible portions of the genome from silent chromatin (see Additional file 1: Figure S14). We therefore assessed whether this may be a general property of NFI-associated boundaries. From 29,158 boundaries of H3K27me3 modifications, 4,687 also featured an H3K36me3 modification boundary (Table 1). Among the H3K27me3 and H3K36me3 overlapping boundaries, 148 (3.15%) were bound by NFI, while just 45 (0.96%) co-localized with a control dataset of 4,794 randomly selected genomic loci. This indicated that the association of NFI with the double boundaries does not result from fortuitous coincidence.

**Table 1:** Distribution of NFI-C-occupied sites at H3K27me3 and H3K36me3 boundaries

**29,158 H3K27me3 boundaries, subdivided in:**	**Matching NFI-occupied site**	**Matching random genomic sites**	**P-value (binomial)**
4,687 H3K27me3 boundaries co-localized with H3K36 boundaries	148 (3.15%)	45 (0.96%)	1.51e-14
2,032 H3K27me3 and H3K36me3 boundaries co-localized with TSS	35 (1.72%)	6 (0.29%)	7.84e-07
1,507 H3K27me3 and H3K36me3 boundaries co-localized with TES	21 (1.39%)	11 (0.72%)	0.050
1,148 H3K27me3 and H3K36me3 boundaries not co-localized with TES or TSS	92 (8.01%)	28 (2.43%)	1.11e-09
24,471 H3K27me3-only boundaries (not co-localized with H3K36 boundaries)	284 (1.16%)	253 (1.03%)	0.167
3,386 H3K27me3-only boundaries co-localized with TSS	67 (0.19%)	19 (0.05%)	5.29e-08
2,153 H3K27me3-only boundaries co-localized with TES	20 (0.09%)	13 (0.06%)	0.162
18,932 H3K27me3-only boundaries not co-localized with TES or TSS	197 (1.04%)	221(1.16%)	0.889

29,158 H3K27me3 modification boundaries were mapped over the murine genome using the ChIP-Part algorithm, and this dataset was intersected with the H3K36me3 boundaries to yield co-localized or non-overlapping boundaries datasets. Boundaries were defined as co-localized if the sequences 2.5 kb up- and down-stream from each boundary overlap by at least 1bp. Colocalization of NFI with the H3K27me3 and H3K36me3 boundaries, or H3K27me3-only boundaries, was assessed using the set of 4,794 occupied sites or using the control set of 4,794 randomly selected genomic sequences. The percentages indicate the fraction of the particular dataset indicated in the left column that overlaps either an NFI site or a randomly selected site.

We next assessed whether the NFI-bound boundaries are necessarily associated to the initiation or termination of transcription. Among the 148 NFI-bound H3K27me3 and H3K36me3 boundaries, 35 (23%) overlapped a TSS while 21 (14.1%) overlapped a transcription end site (TES). Thus, the majority (62.9%) of these NFI-bound double boundaries did not map to known transcriptional initiation or termination sites (92 out of 148 double boundaries, Table 1). From the 1,148 double boundaries that are not localized at known TSS or TES, 92 (8.01%) were bound by NFI, whereas only around 2.4% were found to occur by random coincidence. This finding was specific of the H3K27me3 and H3K36me3 double boundaries, as a similar analysis of H3K27me3-only boundaries that do not overlap TSS or TES did not reveal an enrichment of NFI as compared to randomly selected genomic sites (1.04% vs. 1.16%, Table 1). We therefore conclude that most NFI-associated H3K27me3 and H3K36me3 boundaries cannot be simply ascribed to chromatin modifications elicited by mRNA transcription, and that over 90 of these double boundaries can result from NFI binding alone. In the case of the Blcap and Nnat gene locus, the H3K36me3–rich chromatin domain consists mostly of DNA downstream of the Blcap gene (Figure 4E). This downstream portion of the locus has been shown to consist of a non-transcribed DNA region that epigenetically regulates the methylation of the Nnat promoter and its paternal allele- and tissue-specific expression [46,47]. Whether some of the 92 NFI-bound double boundaries may correspond to as yet unknown imprinted loci remains to be evaluated.

It has been proposed that physical connections of the chromatin to the nuclear lamina may mediate boundaries between open and closed chromatin structures in the interphase nucleus [48,49]. Thus, we tested whether the anchoring locations of DNA to the nuclear lamina may also be enriched in *in vivo* occupied NFI sites, using DNA interactions data for the lamin B1 component of nuclear lamina in human fibroblasts. Genomic coordinates of boundary regions, featuring transitions from the nuclear lamina to the interior of the nucleus, were converted to the mouse genome assembly mm9 using the liftOver tool from UCSC. Out of 2,688 nuclear lamina boundaries, 2,470 could be successfully mapped to the equivalent genomic locations on the mouse genome. NFI binding occurred within 2.5 kb of 85 (or 3.4%) of the nuclear lamina boundaries, and the occupied sites were located predominantly over the lamin-poor side of the nuclear lamina boundaries. However, unlike histone modification boundaries, the occupied NFI sites were not specifically enriched at the boundary position, as they occurred generally over the whole 100kbp of nuclear lamina-free genomic regions extending away from the boundary (Figure 4D). Thus, the occurrence of NFI at genomic boundaries correlates with specific histone modifications that contribute to gene expression regulation but not with structural boundaries involving the nuclear lamina.

### Active promoters contain preferred sites for NFI binding

**Figure 5:**
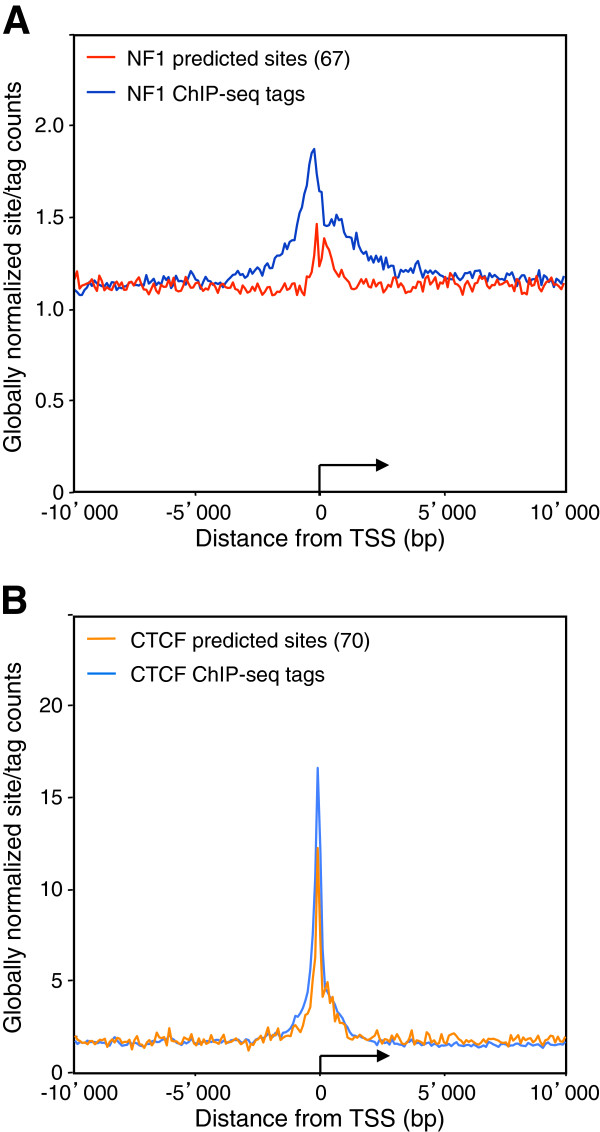
**Increased NFI promoter association does not correlate with high predicted affinity.** Average NFI (panel **A**) or CTCF (panel **B**) ChIP-Seq tag counts were calculated in windows of 100 bp for regions 10kb up- and down- stream of the RefSeq annotated transcription start sites (TSS). TSS were oriented and the broken arrows indicate the initiation site and the direction of transcription. Tag counts were normalized globally, as a fold increase over the genome-wide average tag count in a window of 100bp. NFI and CTCF predicted site counts were normalized similarly as a fold increase over the genome average site count in a window of 100 bp using the indicated weight matrix cut-off scores.

The finding that the NFI-occupied binding sites often coincide with TSS prompted us to analyze whether particular promoters may preferentially associate with this transcription factor and whether it may be linked to promoter activity. NFI ChIP-Seq distribution showed a 2-fold enrichment on the collection of all RefSeq transcription start sites on the mouse genome, while predicted NFI sites were not over-represented at promoters, except for very weak predicted sites (matrix threshold 67) which showed a slight overrepresentation at TSS (Figure 5A). This contrasts the CTCF transcription factor, which showed a similar enrichment of both ChIP-Seq tags and high score predicted site counts close to TSS (Figure 5B). This indicated that NFI binding sequences are not significantly enriched at mouse promoters as compared to intergenic and transcribed regions, but that it is the association to the binding sites *per se* that occurs preferentially at promoters. This may possibly result from synergistic interactions with other transcription factors and/or more permissive chromatin structures that may occur at promoter regulatory sequences, allowing NFI to interact with weak sites. Consistently, NFI sites predicted to occur within 2 kb of a TSS with a moderate to high affinity, with a weight matrix threshold of 85, were associated to approximately 2-fold more ChIP-Seq tag counts than those mapping away from TSS. Thus, we concluded that NFI binding is favored at TSS for poorly- as well as for well-conserved binding sequences.

**Figure 6:**
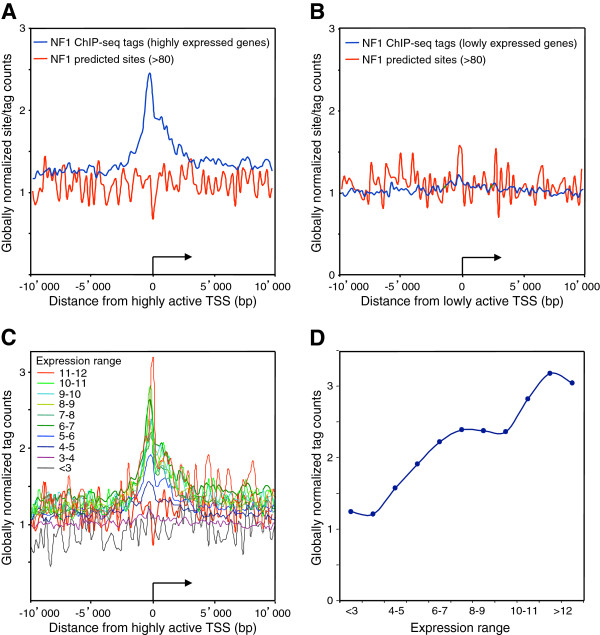
**NFI tag count is increased near transcription start sites of highly-expressed genes.** Average NFI ChIP-Seq tag counts were calculated in windows of 200 bp for regions 10 kb up- and down- stream of the RefSeq annotated transcription start sites (TSS). TSS were oriented and the broken arrows indicate the initiation site and the direction of transcription. Tag counts were normalized globally, as a fold increase over the genome average tag count in a window of 200 bp. NFI predicted sites were mapped on the reference mouse genome mm9 with a matrix cut-off score 67. The NFI predicted site count was normalized in the same way, as a fold increase over the average site count in a bin window of 200 bp. (**A**) Highly-expressed genes with Affymetrix expression levels greater than 8. (**B**) Low- or non- expressed genes with Affymetrix expression levels lower than 4. Genes were separated into 11 groups according to their Affymetrix expression level (<3, 3–4, 4–5, 5–6, 6–7, 7–8, 8–9, 9–10, 10–11, 11–12, >12). (**C**) For each of these groups, the average NFI ChIP-Seq tag counts was calculated in windows of 200 bp for regions 10 kb up- and down- stream of the RefSeq annotated transcription start sites (TSS). Tag counts were normalized globally, as a fold increase over the genome average tag count in a window of 200 bp. (**D**) Peak maxima for each of the gene groups found at the TSS was plotted against the expression level.

We next assessed whether promoter activity may correlate with NFI binding using microarray gene expression data from wt C57Bl6 (wt) and NFI-C ko MEF cells [50]. 3115 highly expressed genes (relative expression level >8) and 4311 lowly or non-expressed genes (relative expression level <4) were selected from mRNA-profiling data from NFIC-expressing cells. The group of lowly or non-expressed genes did not show significant association with NFI near their TSS, while highly expressed genes showed the asymmetric profile seen for the total set of genes, with a slight preference for the transcribed portion of the genes (Figure 6A and 6B). As predicted sites were not overrepresented at the TSS of either class of genes, we concluded that NFI is preferentially bound to active promoters. Thus, transcriptional activity may favor NFI binding through synergistic interactions and/or a permissive chromatin structure. Alternatively, but non-exclusively, the transcriptional activity of these promoters may result at least in part from NFI binding. However, the latter possibility would leave open the question as to what would be driving NFI to bind to and to activate this subset of promoters in the first place.

### NFIC as an activator of gene transcription

The association of NFI with H3K4me3- and H3K36me3-enriched domains suggested that NFI may mediate gene expression activation. To test this hypothesis, we first divided mouse genes into 11 categories according to their expression levels in MEF cells. NFI tag counts were enriched over the promoters of highly expressed genes, while it decreased following a linear trend towards the group of the lowest expressed genes (Figure 6C and 6D). When the RefSeq annotated genes containing one or more *in vivo* NFI sites bound to their 5 kb upstream regions were singled out from the genes not containing upstream NFI sites (yielding 1,227 out of 21,772 RefSeq genes), NFI-bound genes showed a higher average transcription level than genes not associated to NFI (6.61 vs. 5.85, respectively, p=2.91E-31, two tailed t-test). This indicated that NFI protein family members may act mainly to activate transcription when bound to promoters.

**Figure 7:**
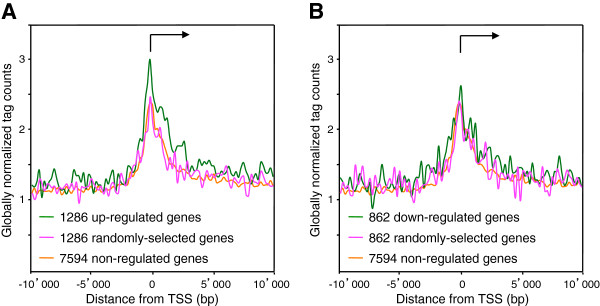
**Transcription start sites of NFI-C up-regulated genes show the highest occupancy with NFI.** (**A**) Genes were considered to be up-regulated by NFI-C if the difference in Affymetrix expression levels between wild-type and NFI-C knock-out cells was greater than 0.5. We found 1286 genes to be up-regulated by NFI-C. As a negative control we used 1286 randomly chosen genes. (**B**) Genes were considered to be down-regulated by NFI-C if the difference in Affymetrix expression level between wild-type and NFI-C knock-out cells was lower than −0.5. We found 862 genes to be down-regulated by NFI-C. As a negative control we used 862 randomly chosen genes. In both graphs we used as a negative control NFI-C non-regulated genes with the change in Affymetrix expression level between −0.05 and 0.05. Average NFI ChIP-Seq tag counts were calculated in windows of 200 bp for regions 10 kb up- and down- stream of the oriented transcriptional start sites (TSS). TSS were oriented and the broken arrows indicate the initiation site and the direction of transcription. Tag counts were normalized globally, as a fold-increase over the genome average tag count in a window of 200 bp.

The transcriptional activation function of the NFI-C family member was assessed from the mRNA profiling data of the wt and NFI-C^−/−^ ko cells, by comparing the group of the 1000 genes most strongly up-regulated by NFI-C to the group of the 1000 most down-regulated genes. The changes of expression levels in these groups indicated that NFI-C activated the expression of up-regulated genes more than it decreased the expression of down-regulated genes (data not shown). NFI-C-regulated genes may be directly bound by NFI-C or indirectly controlled by e.g. the altered expression of NFI-C-regulated transcription factors. Thus, we tested whether the NFI group of proteins would bind preferentially NFI-C regulated genes. As a control group of non-regulated genes, we selected the 7,594 genes whose changes in expression levels were affected by less than 5% when comparing wt and ko cells. As additional control groups, we randomly selected datasets of the same number of mouse genes. NFI tag count was increased 3-fold over background on the TSS of the 1,286 NFI-C up-regulated genes, and 2.6-fold for the group of 862 NFI-C down-regulated genes (Figure 7). The two control groups of NFI-C non-regulated genes showed consistent profiles, and a relatively lower 2.4-fold enrichement of ChIP-seq tags over the TSS. When predicted site occupancy was analyzed over up-regulated, non-regulated or randomly selected genes, a preferential binding to up-regulated genes was also observed (data not shown). Overall, we conclude that NFI more prominently binds the TSS of the up-regulated genes as compared to non-regulated or down-regulated genes, implying that NFI is a direct activator of a significant subset of the up-regulated target genes.

What is the precise role of NFI-C in the up-regulation of promoters bound by NFI family members, and what happens at these promoters in the NFI-C ko cells? One possibility is that these promoters are not bound by any member of the NFI family in the ko cells. Alternatively, they may be bound by less potent NFI activators. To distinguish between these two hypotheses we analyzed predicted site occupancy in the NFI ko cells in the same manner as we did for the wild-type cells (see Additional file 1: Figure S15). Plots obtained from wild-type and ko cells are nearly identical, suggesting that there is no change in overall NFI protein occupancy at NFI sites in up-regulated promoters. This rules out member-specific occupancy of NFI sites as an explanation for the ko phenotype, and strongly favors the hypothesis that different members of the NFI proteins have different regulatory effects when bound to the same *cis*-regulatory DNA element.

Up-regulated genes had the highest density of occupied sites when compared to the genome-wide average density or to non-regulated genes (see Additional file 2: Table S4). Consistently, up-regulated genes showed a higher density of predicted sites than non-regulated genes (20.3 vs. 16.9 sites/Mb, respectively), indicating that preferential binding to up-regulated genes depends at least in part upon a higher density of genomic binding sequences. However, an even higher density of predicted sites was observed on intergenic sequences when compared to up-regulated genes (22.5 vs. 20.3 sites/Mb, respectively), but the occupancy of these sites were lower than that of upregulated genes (5.2 vs. 7.9 sites/Mb, respectively). Thus, we conclude that NFI binding to up-regulated genes must be dictated by both a higher density of high-affinity binding sites and by a more permissive environment in terms of cooperating transcription factors and/or chromatin structure as opposed to intergenic locations in the genome. Non-regulated genes show an intermediate association to NFI (see Additional file 1: Figure S15), indicating that they may be partly permissive for occupation. Overall, these findings imply that the occurrence of binding sites at non-genic locations is not subjected to a negative selection pressure, which can be explained by the fact that these sites are infrequently occupied and thus would not divert these transcription factors from binding target genes. However, binding sites appear to be counter-selected at non-regulated genes, as expected from the observation that genes may have a chromatin structure permissive for NFI binding, and thus would mediate improper regulation of non-target genes, and/or compete for occupancy with physiologically-relevant NFI target genes, should they contain proper binding sequences.

## Conclusions

In this study, we determined the DNA-binding specificity and genome-wide distribution of NFI family proteins, and we find that genomic binding site predictions correlate well with DNA binding site specificity and with the frequency of occupancy in living cells. However, we also find that some high-scoring predicted sites are not occupied, and conversely, that not every *in vivo* site contains a strong predicted site in its vicinity. This indicates that although the *in vitro* or *in vivo* DNA binding sequence specificities are indistinguishable, the actual occupancy of genomic binding sites may be influenced by the genomic context, for instance by potential interactions with other transcription factors and/or by the status of chromatin modifications.

NFI binding to predicted sites was favored at promoters that are enriched with the H3K4me3 chromatin mark of active promoters, and notably at the promoters of genes that are up-regulated by NFI-C. Gene bodies and 3’UTRs are also more populated by occupied NFI sites than intergenic regions. The distribution of NFI in the genome is thus not uniform, while the distribution of predicted binding sites is not biased in this respect.

Previous work suggested that NFI may act as a boundary protein that separates distinct chromatin structures at telomeric loci, and that it may directly interact with nucleosomes on synthetic reporter promoters in transfected cells. Nucleosome positioning on the mouse mammary tumor virus promoter was associated to NFI-mediated chromatin reorganization and promoter activation [22,51]. However, whether NFI may also interact with nucleosomes and/or elicit chromatin domain boundaries within a natural chromosomal context had not been assessed so far. This ChIP-Seq study yielded results consistent with a direct interaction of NFI with nucleosomal particles. This observation is in agreement with previous observations that the NFI trans-activation domain can interact directly with the histone H3.3 variant *in vitro,* a marker of expressed genes and that it alters the interaction of reconstituted nucleosomal cores with DNA [19,52].

These findings also correlate well with another proposed mode of action of NFI, which is to act as chromatin boundary (or barrier) protein that can prevent the spreading of closed chromatin conformation from the telomeres upon subtelomeric positions [24]. Our data indicate that NFI globally associates with chromatin domain boundaries separating permissive and silent chromatin markers, as defined by its co-localization with H3K27me3 and H3K36me3 boundaries of opposite polarities. It has been previously reported that promoters and/or CTCF sites often constitute chromatin domain boundaries [53]. Consistently, we find that some of these NFI-associated boundaries correspond to TSS. However, we also observed NFI association with H3K27me3 and H3K36me3 boundaries that do not map at TSS or TES, as 8% of such boundaries are bound by NFI. This is by far the highest degree of correlation observed at such boundaries, indicating that this may be a prominent function of NFI proteins. The proposed interaction of NFI with nucleosomal particles may provide an explanation for the previous observations that NFI histone-binding domain blocks the propagation of silent chromatin structures at human and yeast telomeres [20,23,24], where this interaction might prevent the self-propagation of histone modifications by the processive association of histone-modifying enzymes to nucleosomes.

Overlaps of functionally distinct chromatin marks have been observed previously, for instance in undifferentiated cells where the co-localization of both open and closed chromatin markers is termed bivalent chromatin [54]. Our results suggest that boundary proteins may specialize in controlling the occurrence of particular combinations of chromatin modifications, as indicated by the finding that NFI associates preferentially with double H3K27 and H3K36 boundaries, but not with simple H3K27 transitions. One such NFI-bound boundary coincides with a regulatory region required for the proper imprinting of the Blcat and Nnat genomic locus, where two overlapping genes are differentially expressed and imprinted during murine and human development [47]. Interestingly, the H3K36me3-rich domain bracketed by NFI binding sites encompasses both these genes as well as a regulatory region that controls imprinting. Furthermore, misregulated expression of the Nnat gene was observed during altered tissue regeneration in NFI-C ko mice, suggesting this locus as a bona-fide regulatory target of NFI-C [50]. Further large scale genomic studies will be required to assess the intriguing possibility that such double boundaries may be indicative of as yet unknown imprinted loci and whether altered imprinting may contribute to some of the developmental and tissue regeneration abnormalities observed in NFI-C knock out mice [30,50,55].

## Methods

### Cell culture

Mouse embryonic fibroblasts (MEF) were extracted from mouse embryos of 14.5 days. Cells from wild-type (WT) and NFI-C knock-out (KO) embryos were cultured in DMEM medium under the following conditions: 37°C, 5% CO2, DMEM (GIBCO, 41966), Supplemental 10% FBS (GIBCO, Fetal Bovine Serum, qualified origin US, 26140–079), 1% v/v nonessential amino-acids (GIBCO, 11140–035), 1% v/v L-glutamine (GIBCO, 25030–024).

### Chromatin Immuno-precipitation (ChIP) and Western blotting

Chromatin was extracted from approximately 20,000,000 cultured MEFs and cross-linked using 11% formaldehyde. Extracted chromatin was fragmented to the average fragment size of 1000 bp using high-frequency sound sonication on VibraCell-75455 (Bioblock Scientific). ChIP was performed as described previously [20], using the commercial antibody against NFI group of proteins (NFI (H300): sc-5567, SantaCruz Biotechnology). Antibody complexes were precipitated using rProtein A Sepharose Fast Flow (Amersham Biosciences). Western blot analysis was performed using the same H300 commercial antibody.

### Illumina/Solexa sequencing

ChIP DNA was processed using the contents of the ChIP-Seq Sample Prep Kit (Illumina). Overhangs were converted into phosphorylated blunt ends, using T4 DNA polymerase, E. coli DNA Pol I large fragment (Klenow polymerase), and T4 polynucleotide kinase (PNK). The 3' to 5' exonuclease activity of these enzymes removes 3' overhangs and the polymerase activity fills in the 5' overhangs. ‘A’ base was added to the 3' end of the blunt phosphorylated DNA fragments, using the polymerase activity of Klenow fragment (3' to 5' exo minus). This prepares the DNA fragments for ligation to the adapters, which have a single ‘T’ base overhang at their 3' end. Adapters were ligated to the ends of the DNA fragments, preparing them to be hybridized to a flow cell. Excess adaptors were removed and a size range of templates was selected to go on the Cluster Station by loading the entire sample on a 2% agarose gel and excising the gel region of 50-400 bp. PCR amplification of the gel-extracted DNA was performed for 18 cycles using adapter-specific primers. Each sample was loaded into 3 separate flow cell channels of the Illumina Cluster Station and then subjected to sequencing-by-synthesis on the Illumina Genome Analyzer sequencing system.

### Read alignment

Alignment of the obtained reads to the mouse reference genome (mm9) was performed using ELAND software (Illumina).

### Data analysis

The ELAND software was used to filter out unmappable tags, as well as those that occur on multiple loci in the haploid genome, and the sequence tag positions were mapped on the reference mm9 mouse genome (July 2007-NCBI37/mm9) allowing up to two mismatches. NFI binding sites were inferred from the mapped sequence tags using the ChIP-Peak software tool from the ChIP-Seq Analysis Server (URL: http://ccg.vital-it.ch/chipseq/chip_peak.php) with the following parameters. As the average size of sequenced genomic DNA fragments was around 260 bp (see Additional file 1: Figure S16), to encompass tags from both ends of NFI binding sites, the tags were counted in a sliding window of 600 bp and we required a minimum distance of 600 bp (“vicinity range” parameters) between two NFI sites to avoid counting the same site multiple times. Tags were shifted 75 bp in their 5’-3’ direction (“centering options”), prior to counting, and we counted only one tag per unique genomic location (“count cut-off” parameter). The tag threshold for defining NFI binding was set between five to ten tags per window, as indicated, from an experimental average tag density of 1 tag / 1204 bp and 1 tag /1291 bp obtained from wt and NFI-C ko cells. For some types of analyses, peaks falling into repeat regions annotated in the UCSC RepeatMasker track were excluded. This was achieved by activating the “Repeat Masker” checkbox on the ChIP-Peak web form.

Correlation analyses of NFI ChIP-Seq tags and NFI predicted sites were performed using the ChIP-Cor tool available on the ChIP-Seq Analysis Server of the Swiss Institute of Bioinformatics (URL: http://ccg.vital-it.ch/chipseq/chip_cor.php). Partitioning of the mouse genome into regions that are rich or poor in a particular histone modification was performed using ChIP-Part tool from the ChIP-Seq Analysis Server (URL: http://ccg.vital-it.ch/chipseq/chip_part.php) using the following parameter settings. Signal rich DNA stretches were defined with a count density threshold of 0.004, the length of DNA stretches was controlled with a transition penalty −20, and the count cut-off of 5 was used to re-set higher tag counts to the cut-off value. Scanning of *in vivo* NFI binding sequences with the NFI weight matrix [29] was done using the OPROF software tool from the Signal Search Analysis server (URL: http://ccg.vital-it.ch/ssa/oprof.php). The comprehensive scan of the mouse genome with the NFI matrix was carried out with fetchGWI software [56]. Genomic coordinates of chromatin and lamina boundaries with the closest internal and external *in vivo* NFI sites are deposited online at GEO database (accession number GSE15844).

Galaxy tools [57] (URL: http://main.g2.bx.psu.edu/) were used to calculate distribution statistics of *in vivo* and predicted NFI sites in mouse intergenic and genic compartments and to create intersections and subtractions of the genomic intervals from different datasets. Galaxy lift-over tool was used to perform lift-over of genomic coordinates of LAD domains from the human genome (version hg18) to the mouse genome (version mm9).

Random sampling of the datasets and creation of the random genomic positions were performed using custom-made C++ scripts, available upon request (Pjanic et al., 2011).

### Motif analysis

MEME software [58] was used to search for over-represented motifs within sets of sequences extracted +/−125 bp from the strongest in vivo sites from wild-type and NFI-C knock-out mouse embryonic fibroblasts. MEME parameters were the following: any number of repetitions per sequence, 5 bp minimum motif width, 20 bp maximum motif width, minimum number of repetitions for each motif - 20, maximum number of repetitions for each motif - 300. Binding sites weight matrices were also generated by hidden Markov training using MAMOT software [59]. The consensus sequence TTGGCNNNNNCGAA was used as starting model. Details of the protocol can be found in [29].

### Datasets repository

NFI ChIP-Seq data from wild type and NFI-C knock-out mouse embryonic fibroblasts, as well as sequenced input control DNA, were deposited at Gene Expression Omnibus (GEO) repository under the accession number GSE15844. Gene expression microarray data for mouse embryonic fibroblasts were taken from GEO repository under the accession number GSE15871. ChIP-Seq data for histone modifications H3K4me3, H3K27me3, H3K36me3 in mouse embryonic fibroblasts were obtained from GEO repository under the accession number GSE12241. Genomic coordinates of lamina associated domains (LAD) from Tig3 human fibroblast cells were downloaded as a publication Supplemental data [49], at the following URL: http://www.nature.com/nature/journal/v453/n7197/suppinfo/nature06947.html. Genomic coordinates of micro RNA precursors in the mouse genome were obtained as a publication Supplemental data [45], at the URL: http://www.cell.com/supplemental/S0092-8674(08)00938-0.

Predicted NFI sites from the mouse genome (ver. mm9) based on the SELEX-SAGE derived position weight matrix [29] are deposited as bed files at the URL: http://ccg.vital-it.ch/BED/CTF-NF1/. Multiple site collections defined with different matrix cut-offs ranging from 67 – 90 score units are provided for each chromosome.

## Competing interests

The authors declare that they have no competing interest.

## Authors’ contributions

MP carried out the ChIPSeq experiments, he performed bioinformatics analysis, together with CDS, GA and PP, under the guidance of PB, and he contributed to writing the manuscript. AG carried out the western blot assays, and GP provided primary embryonic cells and mRNA profiling data. JA, JK and GD analyzed the DNA length of immunoprecipitated protein-DNA complexes. NM supervised the study together with PB, and participated in the design of experimental work and to drafting the manuscript. All authors read and approved the final manuscript.

## Supplementary Material

Additional file 1: Figure S1 Number of predicted genomic NFI binding sites as a function of the position weight matrix score threshold. **Figure S2.** NFI predicted sites in the vicinity of in vivo occupied sites - effect of different tag threshold values for defining in vivo binding sites. **Figure S3.** NFI ChIP-Seq tags preferentially map to the vicinity of NFI predicted sites – effect of lowering the weight matrix score cut-off. **Figure S4.** Comparison of ChiP-Peak and MACS algorithms for peak calling in ChIP-Seq experiments. **Figure S5.** Tags mapping on plus and minus strands are symmetrically distributed around NFI predicted sites. **Figure S6.** Sequence analysis of NFI in vivo sites from wild-type and knock out mouse embryonic fibroblasts. **Figure S7.** NFI-C knock-out mouse embryonic fibroblasts show reduction in NFI protein levels and occupancy of predicted sites. **Figure S8.** Positional correlation between plus and minus tags corresponds to the NFI –DNA complex length. **Figure S9.** Positional correlation of tags mapping on plus and minus strand from unprecipitated control dataset. **Figure S10.** Atomic force microscopy assay of the length of DNA fragments generated by the sonication of crosslinked chromatin. **Figure S11.** Proposed mode of interaction of NFI and nucleosomal particles based on the ChIP-Seq analysis. **Figure S12.** NFI in vivo-occupied sites at miRNA TSS colocalize with H3K4Me3 and H3K36me3 modifications. **Figure S13.** Distribution of distances from histone modification boundaries to the closest NFI or randomly selected site. **Figure S14.** NFI in vivo sites are often located near chromatin domain boundaries. **Figure S15.** NFI predicted sites are more frequently occupied at NFI-C up-regulated genes. **Figure S16.** Average ChIP DNA fragment length submitted for sequencing with the Illumina Genome Analyzer. Click here for file

Additional file 2: Table S1 DNA motifs found with the program peak-motifs in peak lists obtained with different tag thresholds. **Table S2.** Distribution of in vivo and matrix-predicted NFI sites on the mouse genome. **Table S3.** Distribution of NFI in vivo sites surrounding miRNA loci. **Table S4.** Predicted and occupied site distribution at NFI-C-regulated and non-regulated genes. Click here for file
