# Supplementary material for: Nuclear Factor I genomic binding associates with chromatin boundaries
Source: BMC Genomics. 2013 Feb 12;14:99. doi: 10.1186/1471-2164-14-99 (PMC3610271; doi:10.1186/1471-2164-14-99)
Supplement: Additional file 1: Figure S1 — Number of predicted genomic NFI binding sites as a function of the position weight matrix score threshold. Figure S2. NFI predicted sites in the vicinity of in vivo occupied sites - effect of different tag threshold values for defining in vivo binding sites. Figure S3. NFI ChIP-Seq tags preferentially map to the vicinity of NFI predicted sites – effect of lowering the weight matrix score cut-off. Figure S4. Comparison of ChiP-Peak and MACS algorithms for peak calling in ChIP-Seq experiments. Figure S5. Tags mapping on plus and minus strands are symmetrically distributed around NFI predicted sites. Figure S6. Sequence analysis of NFI in vivo sites from wild-type and knock out mouse embryonic fibroblasts. Figure S7. NFI-C knock-out mouse embryonic fibroblasts show reduction in NFI protein levels and occupancy of predicted sites. Figure S8. Positional correlation between plus and minus tags corresponds to the NFI –DNA complex length. Figure S9. Positional correlation of tags mapping on plus and minus strand from unprecipitated control dataset. Figure S10. Atomic force microscopy assay of the length of DNA fragments generated by the sonication of crosslinked chromatin. Figure S11. Proposed mode of interaction of NFI and nucleosomal particles based on the ChIP-Seq analysis. Figure S12. NFI in vivo-occupied sites at miRNA TSS colocalize with H3K4Me3 and H3K36me3 modifications. Figure S13. Distribution of distances from histone modification boundaries to the closest NFI or randomly selected site. Figure S14. NFI in vivo sites are often located near chromatin domain boundaries. Figure S15. NFI predicted sites are more frequently occupied at NFI-C up-regulated genes. Figure S16. Average ChIP DNA fragment length submitted for sequencing with the Illumina Genome Analyzer. [file 1471-2164-14-99-S1.pdf]

A

C	4	-2	-20	-4	6	0	0	0	0	-3	-2	-9	-20	10	10
G	6	-5	-23	-19	10	-4	0	0	0	-4	-2	10	10	2	-1
T	-1	2	10	10	-2	-4	0	0	0	-4	10	-19	-23	-5	6
A	10	10	-20	-9	-2	-3	0	0	0	0	6	-4	-20	-2	4

B

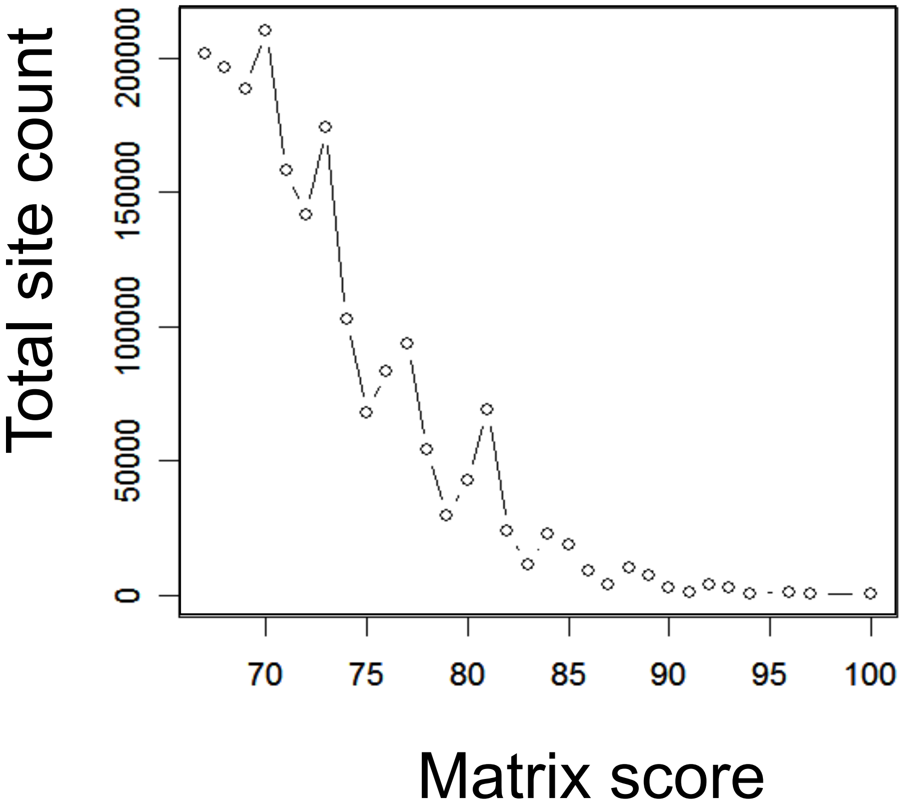

Figure S1. Number of predicted genomic NFI binding sites as a function of the position weight matrix score threshold.

A. NFI position weight matrix, as based on SELEX-SAGE in vitro binding data (Roulet et al., 2002). B. Exponential relation of the number of NFI predicted sites in the mouse genome with the score threshold of the position weight matrix, where the maximum predicted affinity corresponds to a weight matrix score of 100.

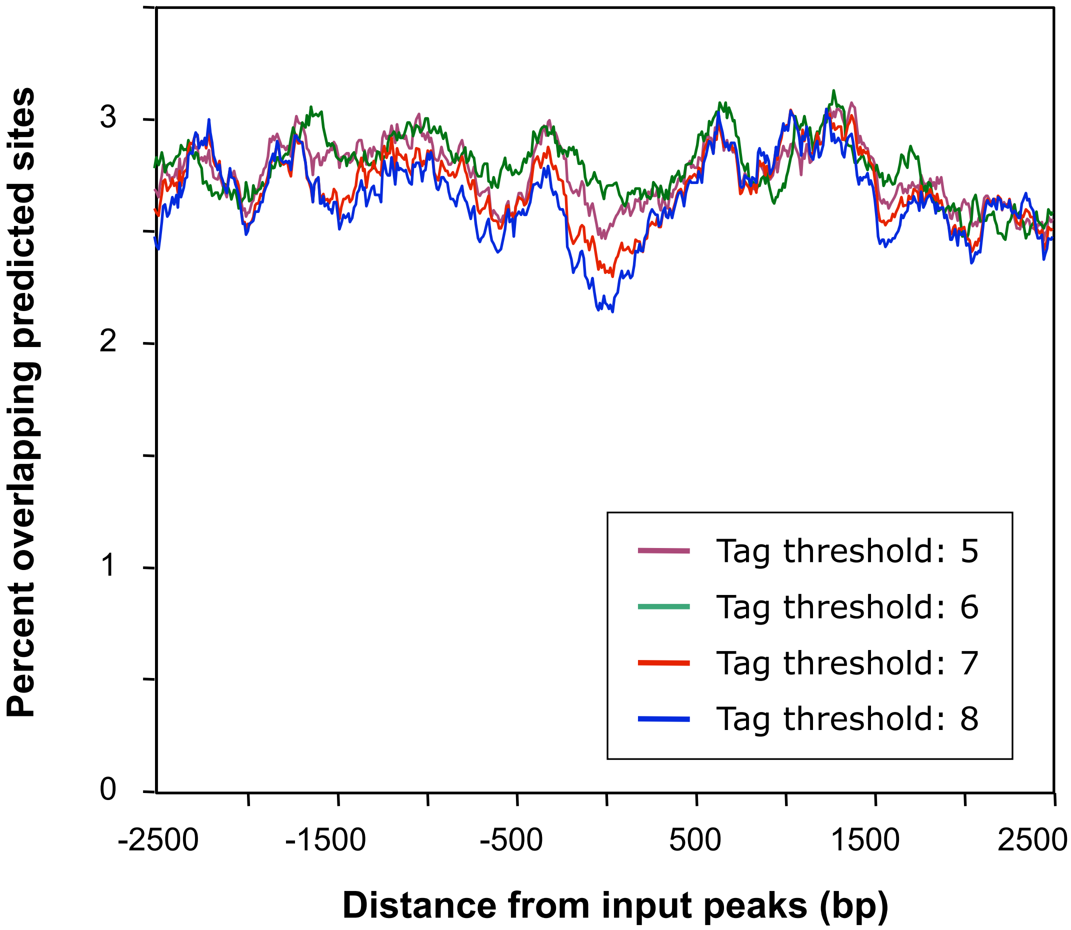

Figure S2. NFI predicted sites in the vicinity of negative control (input) sites.
Input DNA tags were treated as in Fig. 2B using different tag thresholds, and such defined control genomic positions were scanned for the overlap with a predicted site in their +/-2500 bp region. The percentage of in vivo NFI sites matching a predicted site was plotted as a function of the distance to the center of the *in vivo*-occupied sites. NFI predicted sites were defined with the cut-off of 79.

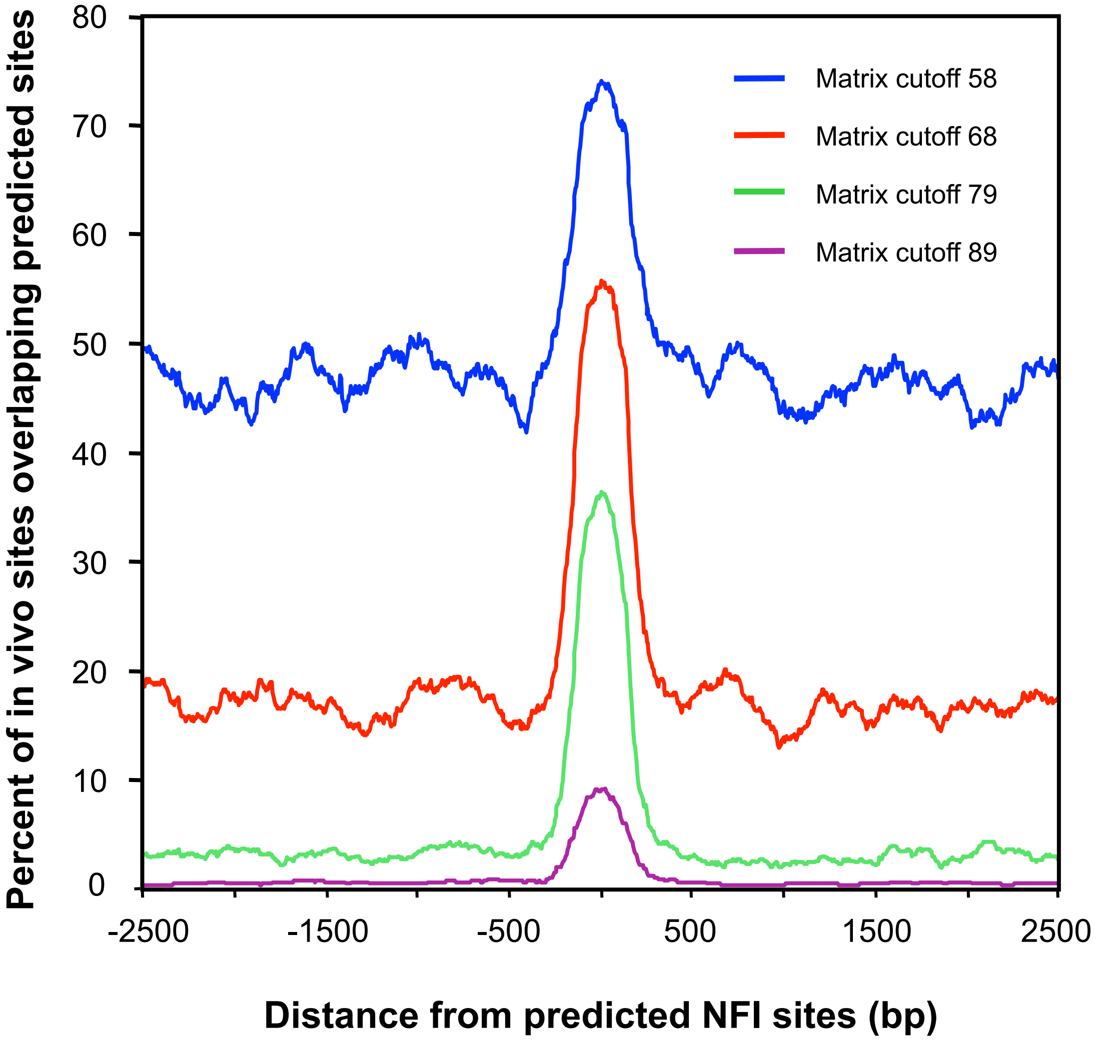

Figure S3. NFI ChIP-Seq tags preferentially map to the vicinity of NFI predicted sites – effect of lowering the weight matrix score cut-off.
Genome-wide scans were performed with collections of NFI predicted sites obtained using a matrix score threshold higher or equal to 89, 79, 68 or 58. The average NFI ChIP-Seq tag counts were calculated in windows of 50bp for a region of 5kb upstream and downstream of the predicted sites. The tag counts were normalized globally as the fold increase over the genome average tag count in a window of 50 bp.

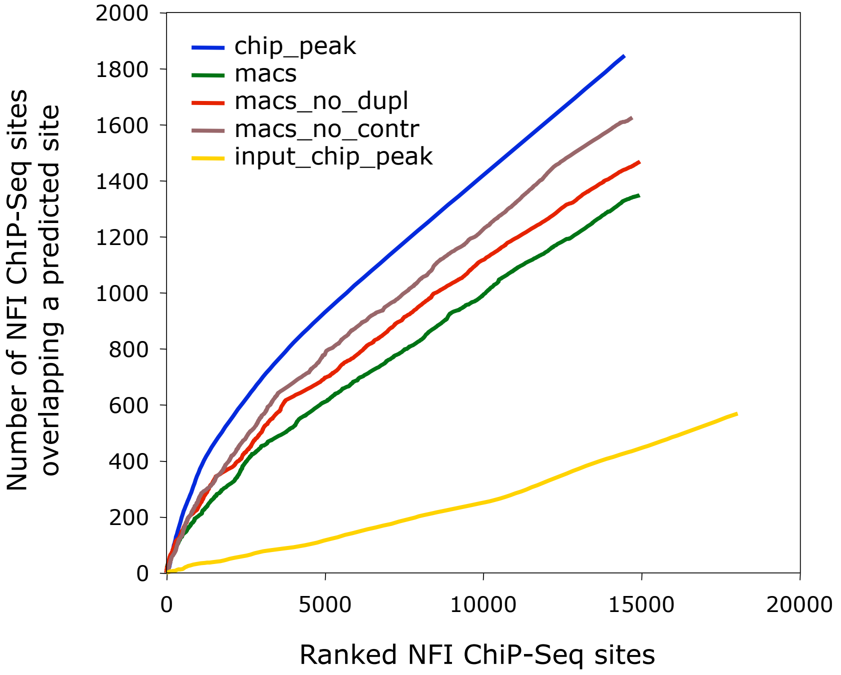

Figure S4. Comparison of ChiP-Peak and MACS algorithms for peak calling in ChIP-Seq experiments

NFI peak lists were constructed using either the ChIP-Peak or MACS algorithm. NFI peak lists created with ChIP-Peak software tool using either wt data set or input genomic DNA data set (labeled 'chip_peak' and 'input_chip_peak') were obtained using default parameters and with a tag threshold of 5. The NFI peak list labeled 'macs' was created using MACS software tool with the following parameters: mfold 5-15, bandwidth 150bp, genome size Mus musculus, allowed duplicate tags, p-value E-3. For the sets 'macs' and 'macs_no_dupl', we included a control data set for normalization purposes. The set 'macs_no_dupl' was generated in the same way as 'macs', but only duplicate tags were counted as a single tag. For the set 'macs_no_contr', no control set was used and MACS parameters were set to call peaks without the control data set while the remaining parameters were equivalent to 'macs_no_dupl'. Overlap of ChIP-Seq sites and predicted sites was considered only if the predicted sites yielded a position weight matrix score equal to or above a threshold of 80.

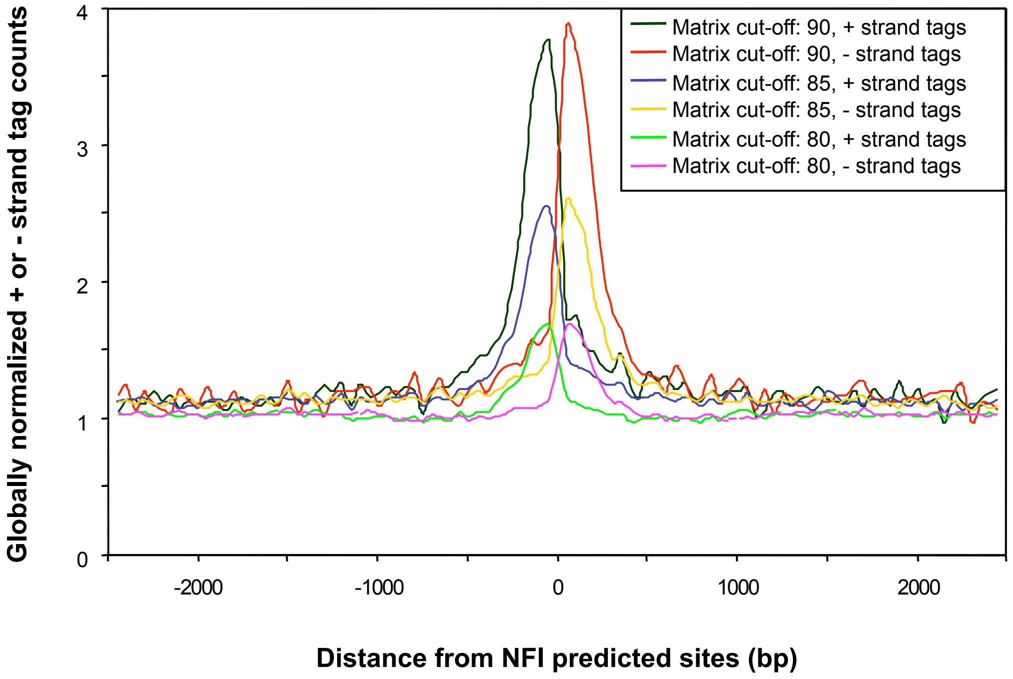

Figure S5. Tags mapping on plus and minus strands are symmetrically distributed around NFI predicted sites

Distribution of NFI ChIP-Seq tags mapping on the plus strand or the minus strand around 12,209 (matrix cut-off 90), 61,492 (matrix cut-off 85) or 231,146 (matrix cut-off 80) NFI predicted sites.

A

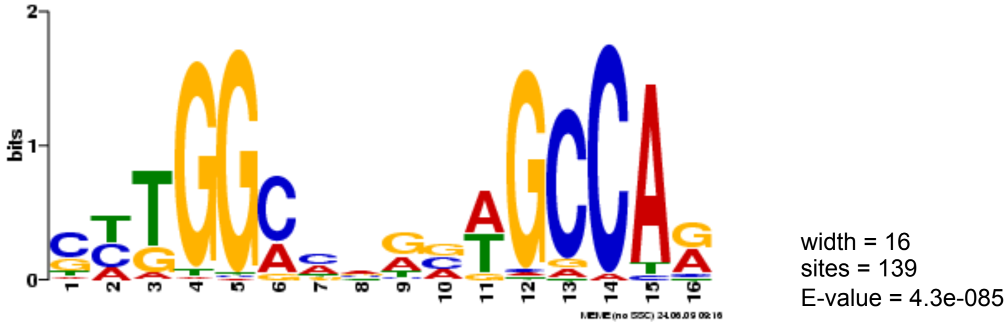

B

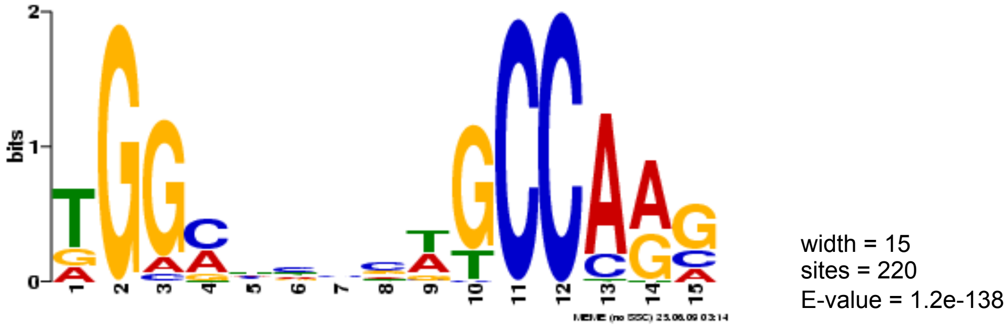

Figure S6. Sequence analysis of NFI in vivo sites from wild-type and knock out mouse embryonic fibroblasts

Sequences encompassing +/-125bp of the 217 strongest NFI in vivo sites from wild-type and NFI-C knock-out mouse embryonic fibroblasts were extracted and analyzed using MEME tool for motif repetitions. (A) Main motif obtained from wild type cells. (B) Main motif obtained from knock-out cells.

Note that opposite strands of the motifs were identified by the MEME algorithm, but that the logo motifs are otherwise related.

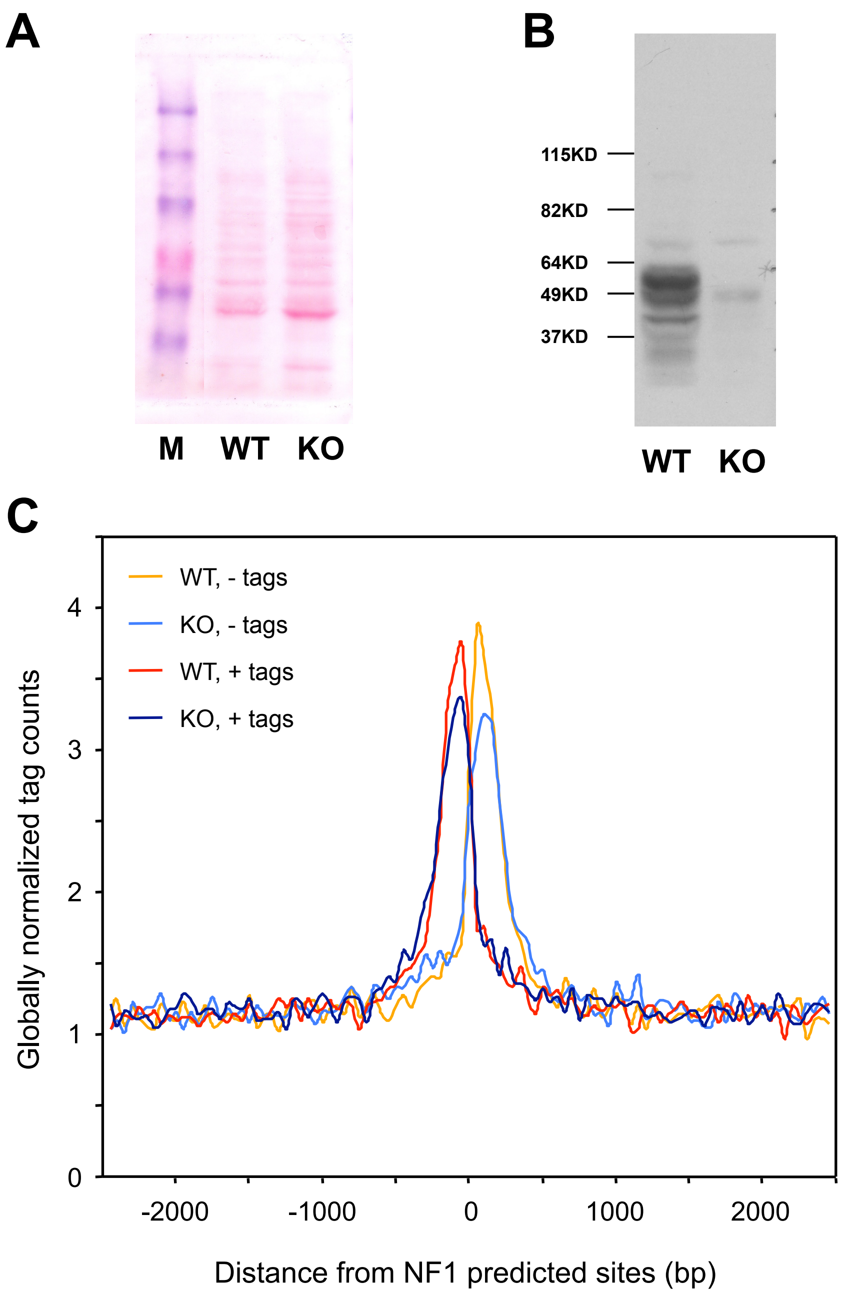

Figure S7. NFI-C knock-out mouse embryonic fibroblasts show reduction in NFI protein levels and occupancy of predicted sites

(A) Ponceau staining of proteins blotted on the nitrocellulose membrane. M – marker, WT – nuclear proteins of wild type mouse embryonic fibroblasts, KO – nuclear proteins of NFI-C knock-out mouse embryonic fibroblasts. (B) Western blot analysis was performed using nuclear extracts from wild type and NFI-C knock-out mouse embryonic fibroblasts. (C) Occupancy of NFI predicted sites. Predicted NFI sites were defined with matrix score >= 90, yielding 12,209 NFI predicted sites. NFI ChIP-Seq tag datasets generated from wild type or knock-out cells were separated according to their mapping to the plus or minus DNA strand. Average NFI ChIP-Seq tag counts were calculated in windows of 50bp for regions 2.5kb up- and down-stream of NFI predicted sites. Tag counts were normalized globally, as a fold increase over the genome average tag count in a window of 50bp.

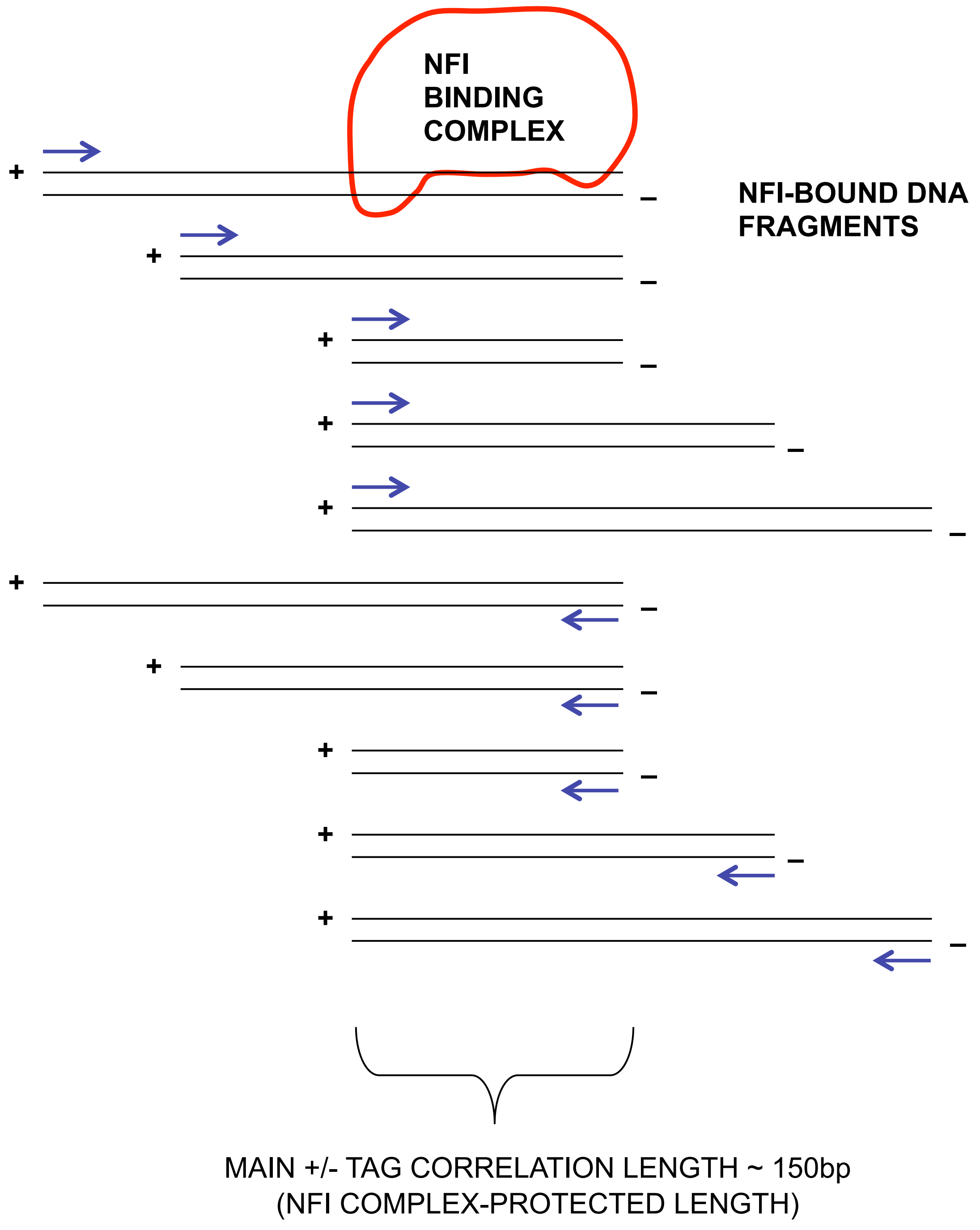

Figure S8. Positional correlation between plus and minus tags corresponds to the NFI –DNA complex length

NFI-DNA complex length could be deduced by positional correlation between the plus and minus strand-mapping tags, revealing a region that is resistant to double-stranded DNA cleavage. Blue arrows represent sequenced tags mapping on either plus or minus strand

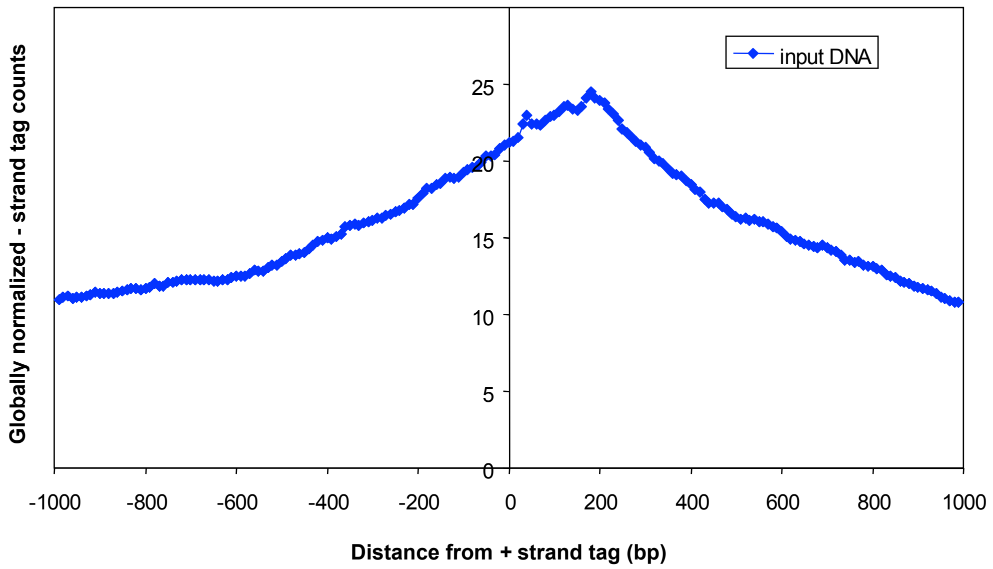

Figure S9. Positional correlation of tags mapping on plus and minus strand from unprecipitated control dataset.

Unprecipitated input DNA tags mapping to the plus strand were centered to position 0 and the distribution of tags mapping on the minus strand is displayed as a function of the distance to the centered plus strand tags, as described in the legend to Figure 2.

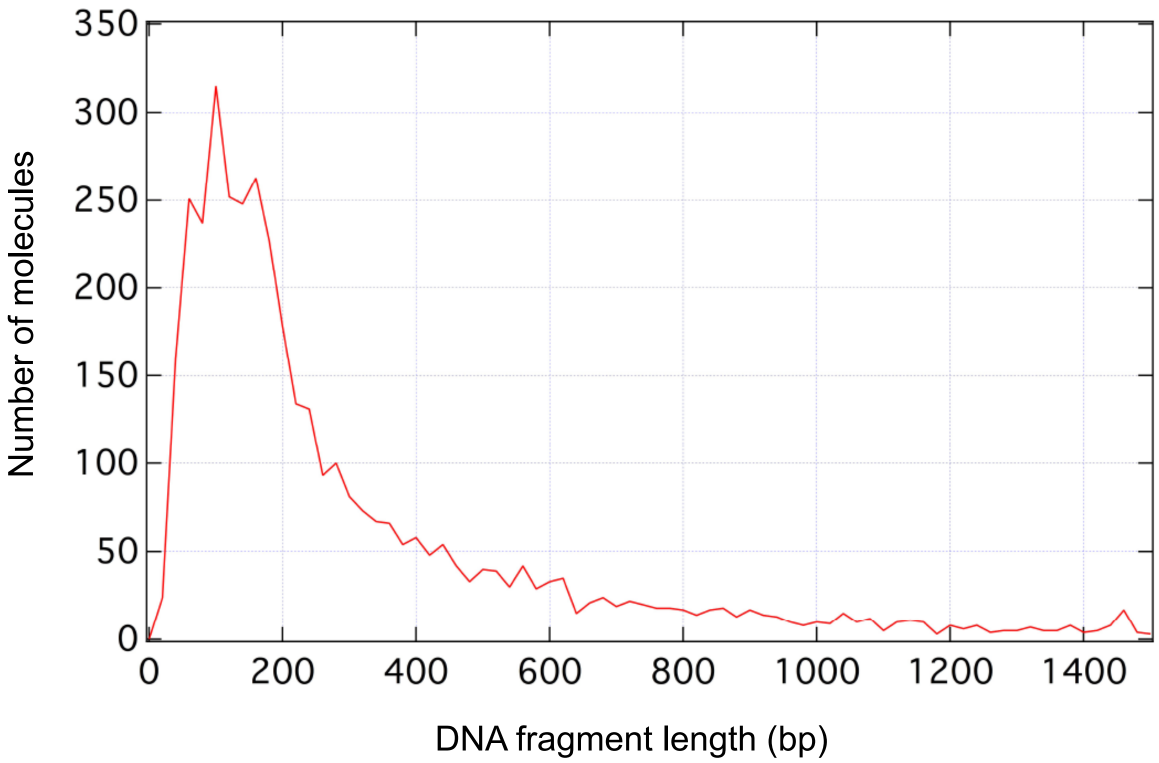

Figure S10. Atomic force microscopy assay of the length of DNA fragments generated by the sonication of crosslinked chromatin.

Unprecipitated input DNA, cross-linked to proteins and fragmented by sonication as for the ChIP-Seq experiments, was released of the cross-linking reactions and spread for length measurements by atomic force microscopy as described by Marek et al., 2005 (Marek J, Demjenova E, Tomori Z, Janacek J, Zolotova I, Valle F, Favre M, Dietler G Interactive Measurement and Characterization of DNA Molecules by Analysis of AFM Images. *Cytometry* **63A**: 87-93).

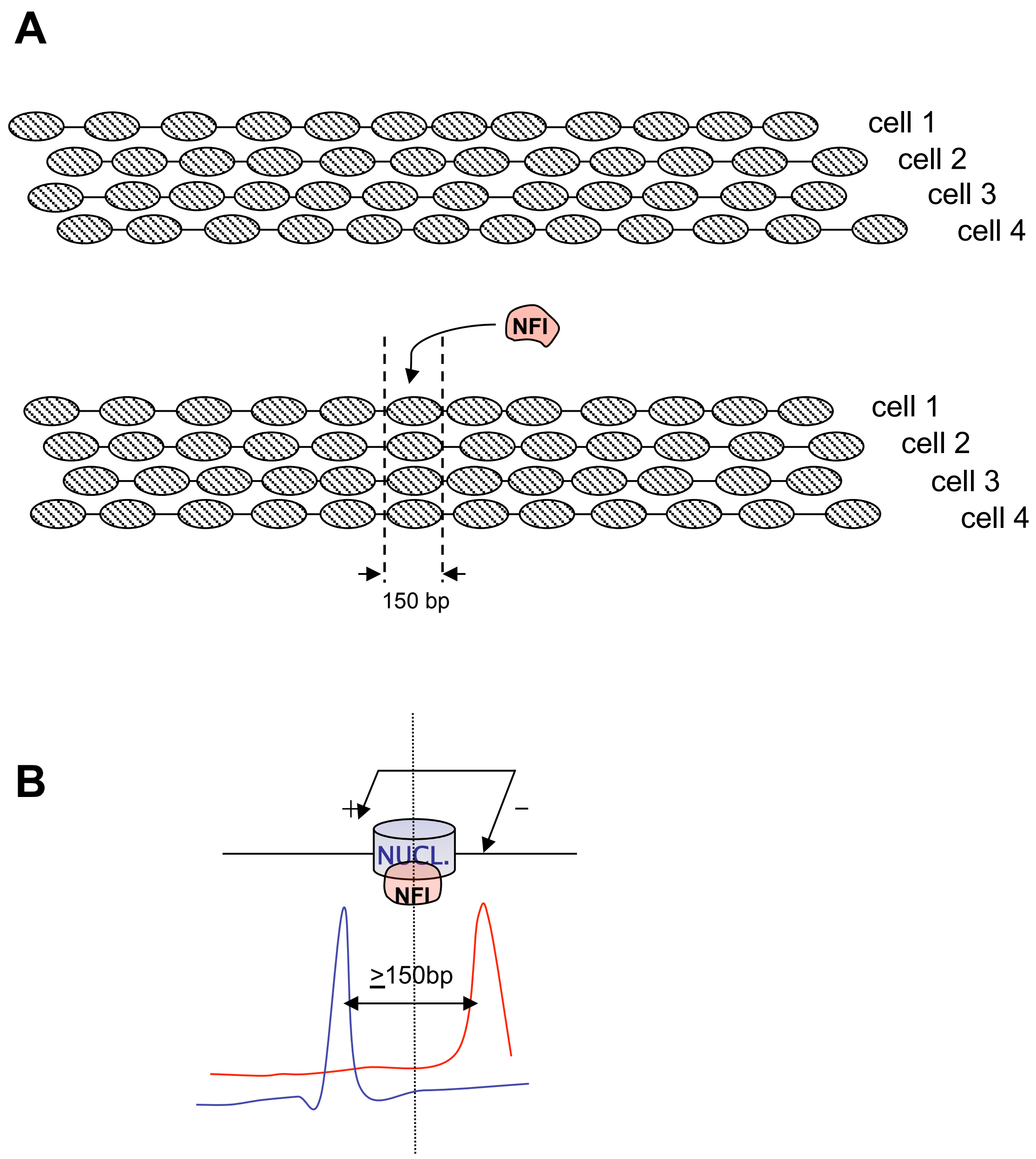

Figure S11. Proposed mode of interaction of NFI and nucleosomal particles based on the ChIP-Seq analysis

(A) Upper drawing: most nucleosomes on the DNA show dynamic translational positioning in different cells of the population. Lower drawing: NFI interacts with a nucleosome that is positioned in all cells of the population that have an occupied binding site, which results in a minimal distance of 150bp between plus and minus strand tags. (B) NFI interacts with the central position of the nucleosome, yielding a symmetrical distributions of plus and minus tags with respect to NFI predicted binding site (showed as dotted vertical line).

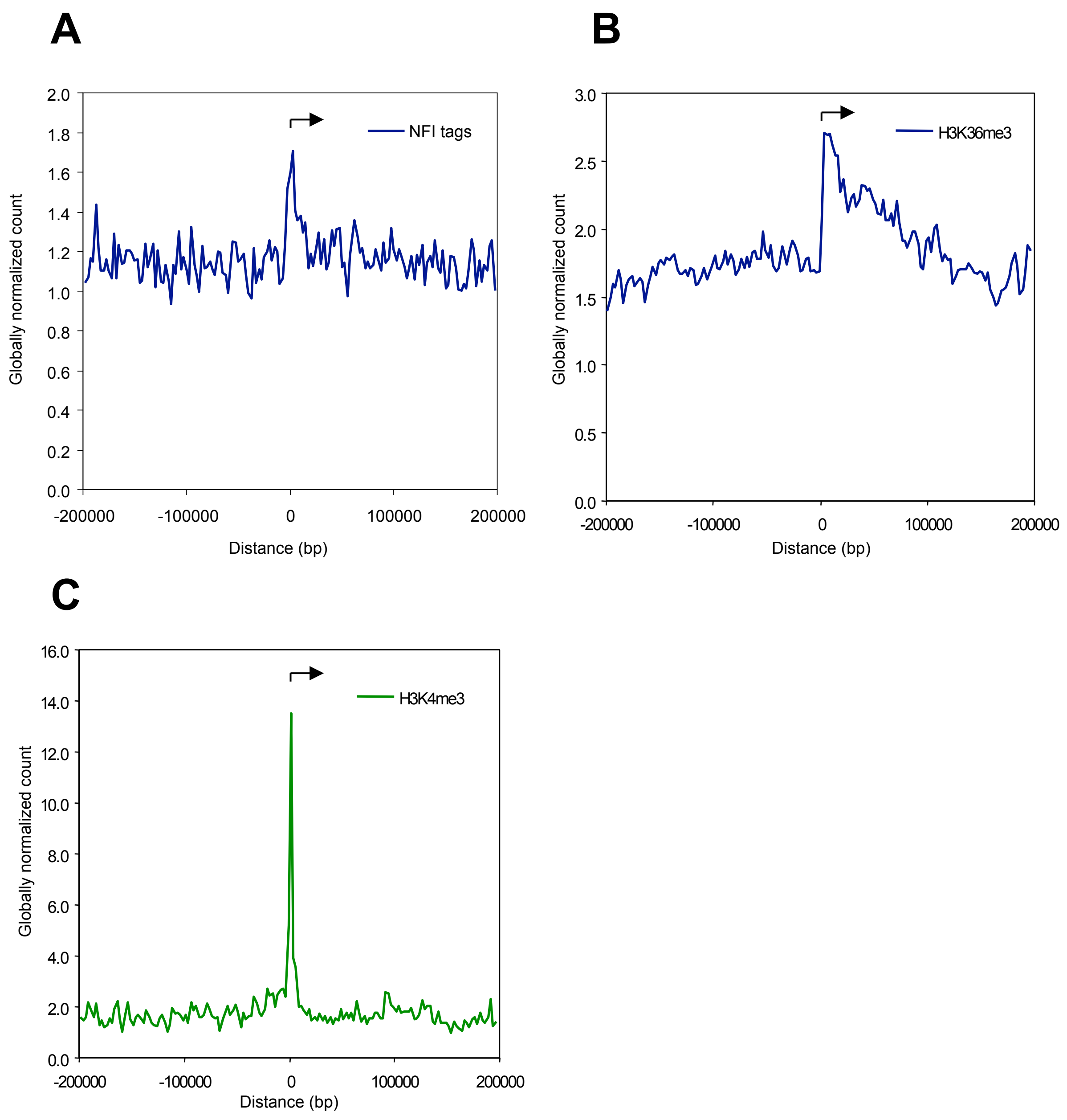

Figure S12. NFI in vivo-occupied sites at miRNA TSS colocalize with H3K4Me3 and H3K36me3 modifications

Predicted transcription start sites (TSS) of 203 annotated microRNA (miRNA) from the mouse genome were oriented in the same direction. Transcription direction is marked with an arrow in each graph. (A) Distribution of NFI ChIP-Seq tags over oriented miRNA TSS. (B) Distribution of ChIP-Seq tags of histone modification H3K36me3. (C) Distribution of ChIP-Seq tags of histone modification H3K4me3.

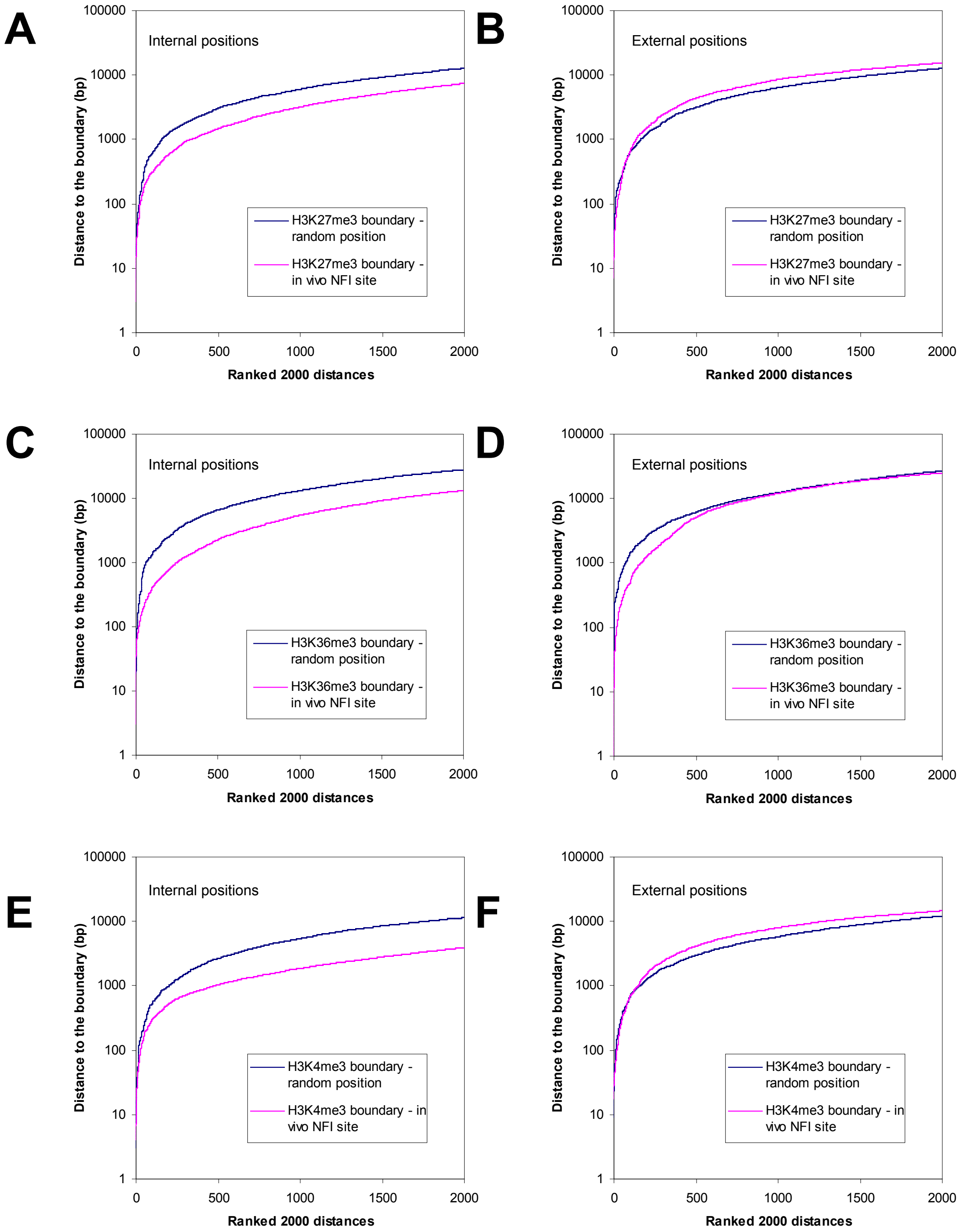

Figure S13. Distribution of distances from histone modification boundaries to the closest NFI or randomly selected site

Distances of histone modification boundaries to the closest NFI or random site were plotted starting from the smallest to the largest values in a log scale. 2000 distances were plotted in every panel. Distances were calculated separately for the external and the internal NFI or random sites. (A) H3K27me3 boundaries, distances to the internal sites. (B) H3K27me3 boundaries, distances to the external sites. (C) H3K36me3 boundaries, distances to the internal sites. (D) H3K36me3 boundaries, distances to the external sites. (E) H3K4me3 boundaries, distances to the internal sites. (F) H3K4me3 boundaries, distances to the external sites.

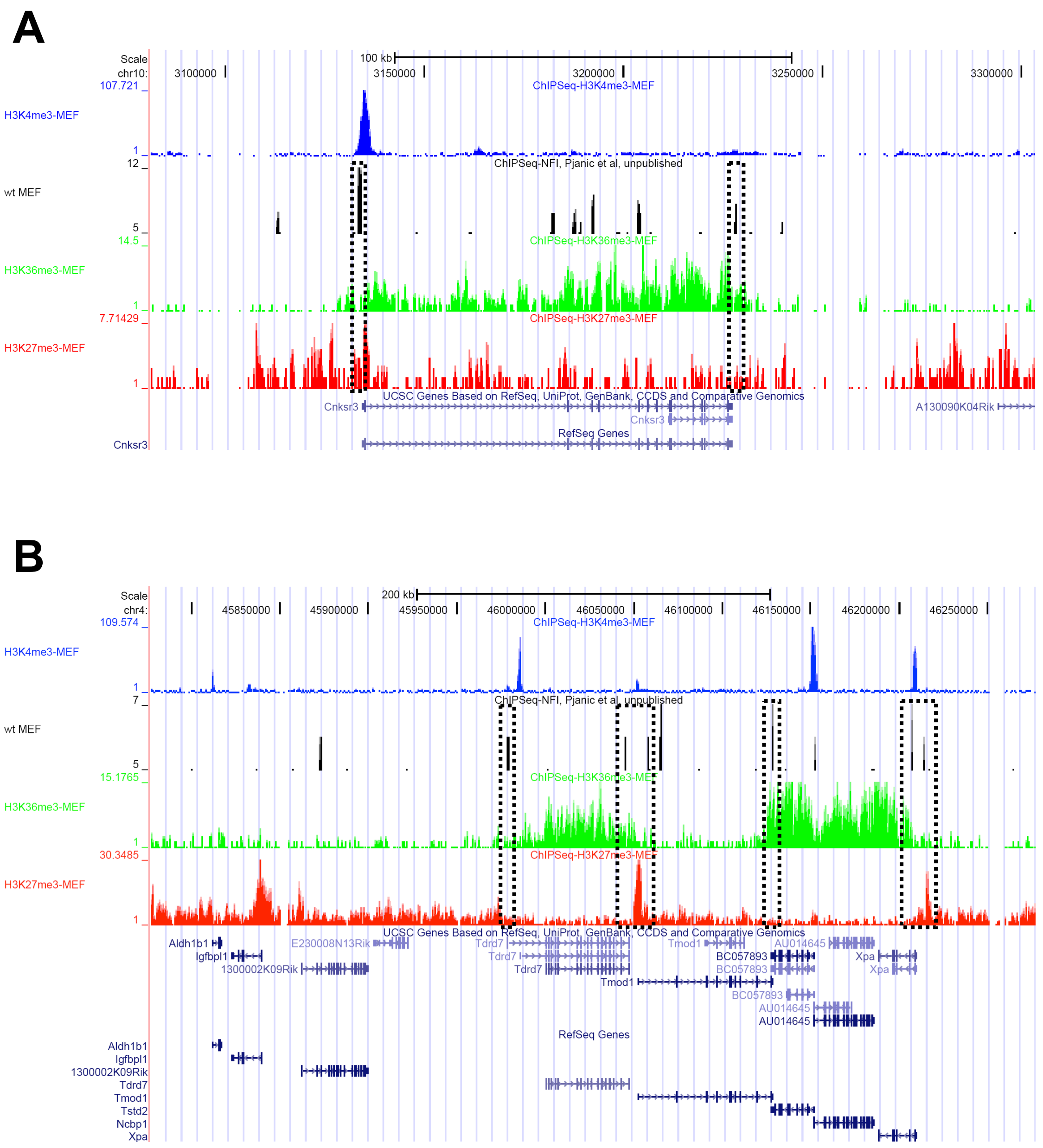

Figure S14. NFI in vivo sites are often located near chromatin domain boundaries

Examples of NFI in vivo binding sites located at the boundaries of open and close chromatin markers, H3K36me3 and H3K27me3, in mouse embryonic fibroblasts. NFI sites at the boundary positions are enclosed with a dotted line. In blue – H3K4me3, in black – NFI binding sites, in green – H3K36me3, in red – H3K27me3. A. NFI binding sites define the region of open chromatin surrounding Cnksr3 gene locus. B. NFI binding sites define the region of open chromatin surrounding the Tdrd7 and Tstd2/Ncbp1/Xpa gene loci.

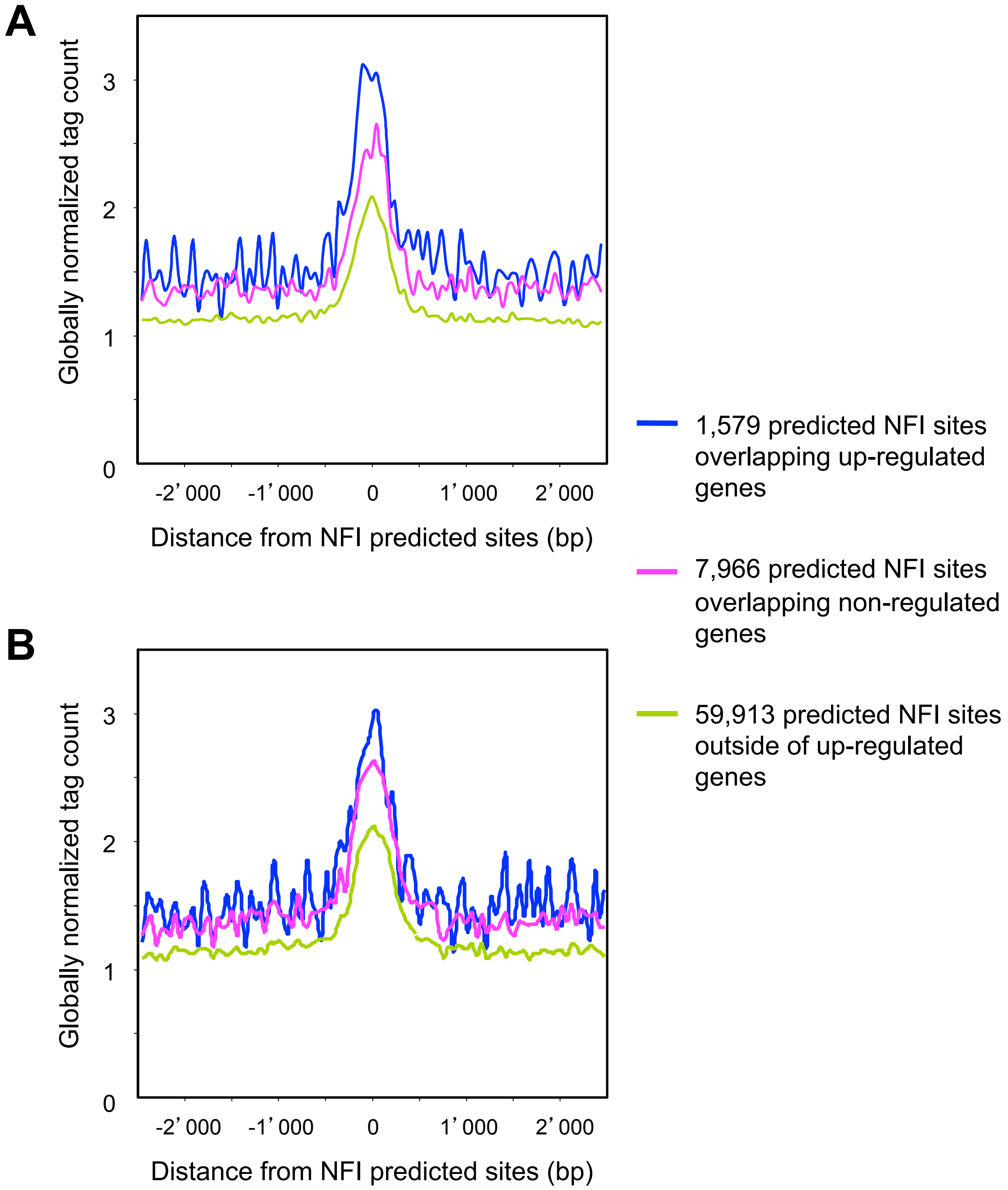

Figure S15. NFI predicted sites are more frequently occupied at NFI-C up-regulated genes
Gene annotations were taken from RefSeq. Gene body regions of NFI-C up-regulated and non-regulated genes were extended to encompass 5kb of upstream and downstream sequences. We then intersected these extended gene regions with NFI predicted sites defined with a matrix score threshold of 85. Out of the total 61,492 predicted sites, 1,579 mapped in the vicinity of up-regulated genes and 7,966 mapped to non-regulated genes. Average NFI ChIP-Seq tag counts were calculated in windows of 50bp for regions 2.5kb up- and down-stream of the NFI predicted sites. Tag counts were normalized globally as the fold increase over the genome average tag count in a window of 50bp. Panel A and B result from ChIP-Seq experiments performed using an antibody recognizing all NFI isoforms on chromatin extracted from either WT cells (A) or NFI-C knock out cells (B), respectively.

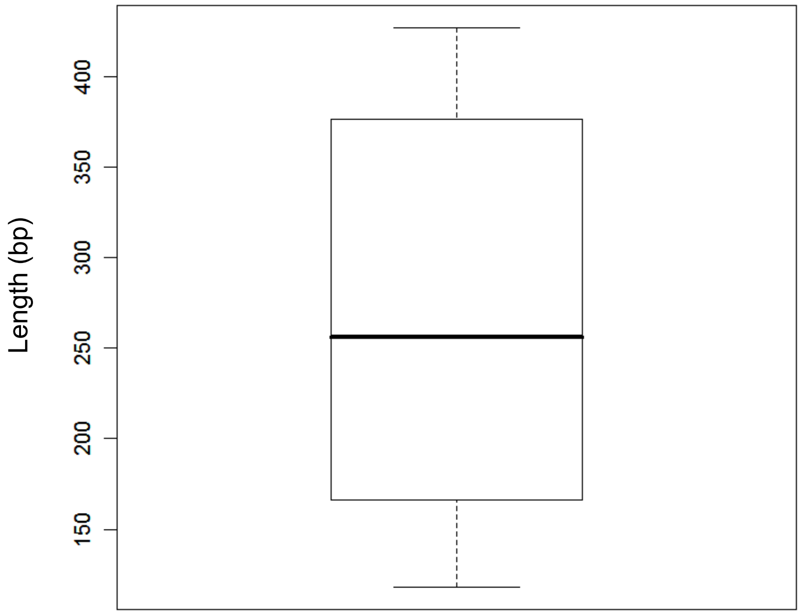

Figure S16. Average ChIP DNA fragment length submitted for sequencing with the Illumina Genome Analyzer.

DNA fragments from ChIP DNA were randomly cloned into TOPO vectors and sequenced. The box-plot represents the distribution of the insert DNA lengths for 19 clones. Sequences from these clones were mapped onto the mouse genome using BLAT, using sequence identity settings comprised between 92% - 100% of the full length DNA fragment. Sequenced fragments size ranged from <100bp up to 400bp, with a median of 256bp.
