# Supplementary material for: Nuclear Factor I genomic binding associates with chromatin boundaries
Source: BMC Genomics. 2013 Feb 12;14:99. doi: 10.1186/1471-2164-14-99 (PMC3610271; doi:10.1186/1471-2164-14-99)
Supplement: Additional file 2: Table S1 — DNA motifs found with the program peak-motifs in peak lists obtained with different tag thresholds. Table S2. Distribution of in vivo and matrix-predicted NFI sites on the mouse genome. Table S3. Distribution of NFI in vivo sites surrounding miRNA loci. Table S4. Predicted and occupied site distribution at NFI-C-regulated and non-regulated genes. [file 1471-2164-14-99-S2.pdf]

Additional file 2.

Table S1. DNA motifs found with the program peak-motifs in peak lists obtained with different tag thresholds

A) Motifs found in peak list obtained with tag threshold 5 (14487 peaks)

**Motif**	**Logo**	**3 Top hits in databases**
oligos_7nt_mkv5_m1 (1790 sites)	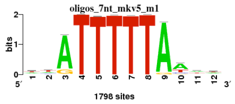	versus jaspar_core_vertebrates: ARID3A,
oligos_7nt_mkv5_m2 (1689 sites)	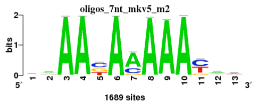	versus jaspar_core_vertebrates: FOXI1,
oligos_7nt_mkv5_m3 (1279 sites)	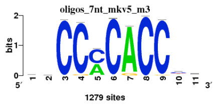	versus jaspar_core_vertebrates: ZEB1, Klf4, ZNF354C,
dyads_m1 (168 sites)	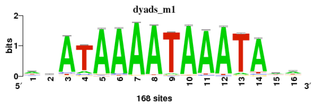	versus jaspar_core_vertebrates: FOXI1, Foxd3,
dyads_m2 (1042 sites)	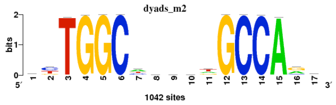	versus jaspar_core_vertebrates: TLX1::NFIC,
dyads_m3 (5377 sites)	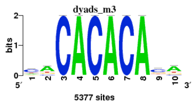	versus jaspar_core_vertebrates: no match

B) Motifs found in peak list obtained with tag threshold 6 (4795 peaks)

**Motif**	**Logo**	**3 Top hits in databases**
oligos_7nt_mkv4_m1 (357 sites)	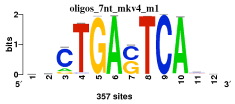	versus jaspar_core_vertebrates: AP1, NFE2L2, NFE2L1::MafG,
oligos_7nt_mkv4_m2 (416 sites)	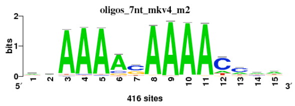	versus jaspar_core_vertebrates: IRF1,
oligos_7nt_mkv4_m3 (604 sites)	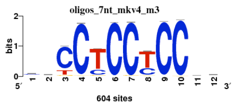	versus jaspar_core_vertebrates: SP1,
dyads_m1 (347 sites)	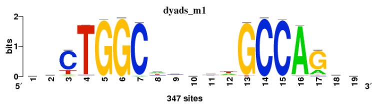	versus jaspar_core_vertebrates: TLX1::NFIC,
dyads_m2 (47 sites)	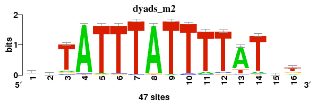	versus jaspar_core_vertebrates: FOXI1, Foxd3,
dyads_m3 (129 sites)	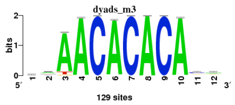	versus jaspar_core_vertebrates: no match

B) Motifs found in peak list obtained with tag threshold 7 (1642 peaks)

**Motif**	**Logo**	**3 Top hits in databases**
oligos_7nt_mkv4_m1 (159 sites)	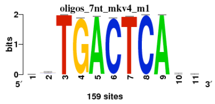	versus jaspar_core_vertebrates: AP1, NFE2L1::MafG, NFE2L2,
oligos_7nt_mkv4_m2 (208 sites)	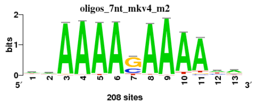	versus jaspar_core_vertebrates: no match
oligos_7nt_mkv4_m3 (177 sites)	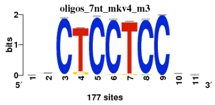	versus jaspar_core_vertebrates: no match
dyads_m1 (8 sites)	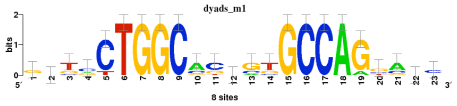	versus jaspar_core_vertebrates: TLX1::NFIC,
dyads_m2 (157 sites)	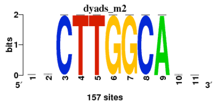	versus jaspar_core_vertebrates: NFIC, TLX1::NFIC,
dyads_m3 (151 sites)	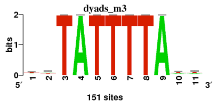	versus jaspar_core_vertebrates: ARID3A,

C) Motifs found in peak list obtained with tag threshold 8 (708 peaks)

**Motif**	**Logo**	**3 Top hits in databases**
oligos_7nt_mkv4 (81 sites)	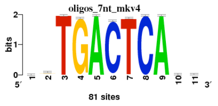	versus jaspar_core_vertebrates: AP1, NFE2L1::MafG, NFE2L2,
dyads_m1 (34 sites)	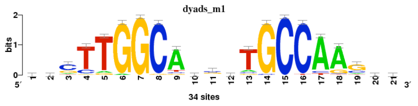	versus jaspar_core_vertebrates: TLX1::NFIC,
dyads_m2 (33 sites)	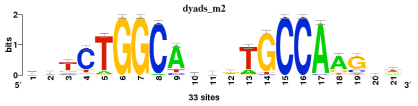	versus jaspar_core_vertebrates: TLX1::NFIC, Hand1::Tcfe2a,
dyads_m3 (49 sites)	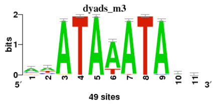	versus jaspar_core_vertebrates: FOXI1, MEF2A, FOXL1,

Sequences within ±100 bp from the peak center positions were extracted from the human genome. The program **peak-motifs** from the RSA-tools (Thomas-Chollier et al. 2011, RSAT peak-motifs: motif analysis in full-size ChIP-seq datasets, Nucleic Acids Res. 40:e31) was used for *de novo* motif discovery with the following options and parameters: discovery modes “over-represented words” and “over-represented word-pairs”, oligomer length 6, number of motifs per algorithms 3, search on both strands.

Table S2. Distribution of in vivo and matrix-predicted NFI sites on the mouse genome

**Total number of sites**	**NFI predicted sites**	**NFI in vivo sites**
Total	61492	4794
Intergenic regions	35054	2138
Upstream (5kb)	2942	428
5'UTR exons	95	47
Coding exons	1404	106
Introns	24348	1966
3'UTR exons	667	73
Downstream (5kb)	3079	232
**Number of sites per Mb**		
Average for the genome	24	1.85
Intergenic regions	20.7	1.26
Upstream (5kb)	27	3.93
5'UTR exons	24.9	12.27
Coding exons	39.5	2.97
Introns	25	2.02
3'UTR exons	29.9	3.26
Downstream (5kb)	28.3	2.13

Table S3. **Distribution of NFI in vivo sites surrounding miRNA loci.**

	**miRNA transcribed regions**	**Upstream 1kb regions**	**Downstream 1 kb regions**
Total bases	10292047	201161	183023
NFI site count	77	3	0
NFI site density	7.4815E-06	1.49134E-05	1.0928E-05
NFI site density per Mb	7.4	14.9	0
Number of miRNAs containing NFI site	43	3	0

In total 203 annotated miRNAs in the mouse genome were used in analysis. Lists of miRNAs that contain NFI sites in either transcribed regions, 1kb upstream or 1kb downstream regions are deposited online at Gene Expression Omnibus (GEO) database under the accession number GSE15844.

Table S4 Predicted and occupied site distribution at NFI-C-regulated and non-regulated genes

	Predicted NFI sites	Occupied NFI sites
Total number of sites	61,492	14,487
Number of sites overlapping NFI-C non-regulated genes (gene bodies and +/- 5kb regions)	7,966	2,412
Number of sites overlapping NFI-C up-regulated genes (gene bodies and +/- 5kb regions)	1,579	617
Number of sites outside NFI-C up-regulated genes	59,913	13,870
Density of sites overlapping NFI-C non-regulated genes (gene bodies and +/- 5kb regions), in number of sites/Mb	16.9	5.1
Density of sites overlapping NFI-C up-regulated genes (gene bodies and +/- 5kb regions), in number of sites/Mb	20.3	7.9
Density of sites outside NFI-C up-regulated genes, in number of sites/Mb	22.5	5.2

For the group of 1286 NFI-C up-regulated genes (difference in expression level >0.5) and the group of 7594 NFI-C non-regulated genes (difference in expression level <0.05) we extended gene body regions for 5kb upstream and downstream. The table shows the total numbers and densities of NFI predicted sites (score >85) and in vivo NFI sites (threshold of 5 tags) mapping in the gene body and +/- 5kb regions of NFI-C non-regulated genes, up-regulated genes and outside up-regulated genes. Densities are expressed in average binding sites per Mb.
